# Supplementary material for: What are the experiences of seeking, receiving and providing FGM-related healthcare? Perspectives of health professionals and women/girls who have undergone FGM: protocol for a systematic review of qualitative evidence
Source: BMJ Open. 2017 Dec 14;7(12):e018170. doi: 10.1136/bmjopen-2017-018170 (PMC5736050; doi:10.1136/bmjopen-2017-018170)
Supplement: Supplementary file 1 [file bmjopen-2017-018170supp001.pdf]

Appendix 1: Detailed Search Strategy

The search strategy was developed in order to meet the requirements for both systematic reviews:

- **R1-**To explore the experiences of FGM-related healthcare across the life course for women and girls who have undergone FGM.
- **R2-**To explore the views on, and experiences of, all cadres of health professionals in providing care for women/girls who have undergone FGM.

Search strategy in Ovid Medline

#	Search terms	Notes
1.	(exp Circumcision, Female/ or (exp Genitalia, Female/ and exp Medicine, Traditional/))	
2.	("female genital mutilation" or "female circumcision" or "female genital cutting" or "traditional female genital surgery" or infibulat* or reinfibulat* or defibulat* or clitoridect* or ((((ritual* or traditional* or ceremon* or sociali#ation*) adj3 (practic* or cut or cutting or surg*)) or circumcis* or excis*) adj3 (female* or wom#n or girl* or child* or adolescen*))).mp.	Different FGM-related terms
3.	((FGM or FGC or TFGS or FGMC or "FGM/C" or thara or tahar or khitan or khifad or khafad or megrez or absum or mekhnishab or kutairi or ibi or ugwu or sunna or bondo or sonde or gudiniin or halalays or qodiin or tahoor or bagne or gadja or ganza or "fanadu di mindjer" or niaka or kuyango or "musolula karoola").mp. or (egypt* or sudan* or ethiopi* or somali* or djibouti* or eritrea* or burkin* or guinea* or sierra leone or leonean* or mauritan* or mali or malian*).ti,ab.) adj3 (female* or wom#n or girl* or child* or adolescen*)	Expanded FGM terms (to include country specific name for the practice.
4.	1 or 2 or 3	
5.	"Emigrants and Immigrants"/ or Refugees/ or "Transients and Migrants"/ or "Emigration and Immigration"/ or exp Vulnerable Populations/ or exp Ethnic Groups/ or 31. exp african continental ancestry group/ or exp asian continental ancestry group/	Immigrant populations
6.	Limit 5 to female	
7.	(immigrant* or immigrat* or migrant* or migrat* or emigrant* or emigrat* or emigre* or expat* or ex-pat* or transients or newcomer* or new-comer* or alien* or incomer* or in-comer* or refugee* or (asylum adj seek*) or asylee* or (refused adj3 (asylum* or refugee*)) or (displaced adj person*) or exile* or “new arrival*” or (country adj2 (birth or origin)) or transnational* or foreigner* or (foreign adj (born or citizen* or national* or origin*)) or (non adj (citizen* or native*)) or ((adoptive or naturali#ed) adj (citizen* or resident*)) or overstay* or trafficked or "spousal migrant*" or ethnic* or ethno* or race or racial*).mp. or ((vulnerab* or disadvantag* or minorit*) adj3 (individ* or person* or people* or population* or communit* or group*)).ti,ab.	Expanded immigrant terms commonly used in OECD countries
8.	Limit 7 to female	
9.	("Black and Minority Ethnic" or "Black & Minority ethnic" or BME or BAME or black african*).mp. or<br>(africa* or "middle east*" or asia* or iran* or iraq* or israel* or oman* or "united arab emirat*" or UAE or "saudi arabia*" or palestin* or india* or indonesia* or malaysia* or pakistan* or egypt* or yemen* or sudan* or ethiopi* or somali* or djibouti* or eritrea* or kenya* or uganda* or	Expanded immigrant terms to include countries and populations

	tanzania* or camero?n* or chad* or niger* or benin* or togo* or ghana* or burkin* or ivory coast* or cote d'ivoire or liberia* or guinea* or sierra leone or leonean* or gambia* or senegal* or mauritan* or mali or malian* or "sara subgroup" or ngama).ti,ab.	where FGM is a common practice
10.	Limit 9 to female	
11.	6 or 8 or 10	
12.	exp Community Health Workers/ or exp Volunteers/ or exp Occupational Groups/ or exp Health Personnel/ or exp Midwifery/ or exp General Practitioners/ or exp Physicians/ or exp Nurses/ or exp Nurses, Community Health/ or exp Nurses' Aides/ or exp Nursing Staff, Hospital/ or exp Nursing Staff/	Health professional cadres
13.	(pregnan* or birth* or childbirth* or matern* or gyn#e* or obstetric* or menstru* or labo#r* or vulv* or vagina* or uter*).ti,ab.	Commonly reported in regard to FGM
14.	exp Pregnancy/ or exp Pregnancy Complications/ or exp Pregnancy High Risk/ or exp Obstetric Labor Complications/ or Female Urogenital Diseases/ or exp Menstruation Disturbances/ or exp Genital Diseases, Female/ or exp Vaginal Diseases/ or exp Vulvar Diseases/ or exp Vaginismus/ or exp Vulvodynia/ or exp Dyspareunia/ or exp Vaginitis/ or exp Vulvovaginitis/ or exp Vaginal Fistula/ or exp Rectovaginal Fistula/ or Genital Diseases, Female/ or exp Infertility, Female/	FGM-related conditions, problems, complications
15.	((pelvic* or back* or urolog* or urogenit* or urinat* or genit* or abdomin*) adj3 (poor* or adverse or complicat* or difficult* or disorder* or dysfunction* or disease* or pain* or risk* or danger* or problem* or issue* or concern* or infect* or inflamm*) adj3 (female* or wom#n or girl* or child* or adolescen*)).mp.	
16.	exp Pelvic Inflammatory Disease/ or exp Urologic Diseases/ or exp Pelvic Pain/ or exp Sexual Dysfunctions, Psychological/ or exp Pelvic Infection/ or exp Kidney Diseases/ or exp Urinary Fistula/ or exp Rectal Fistula/ or exp Urinary Bladder Fistula/	
17.	Limit 16 to female	
18.	((anxiet* or anxious* or depress* or self-harm* or self-injur* or suicid*) adj3 (female* or wom#n or girl* or child* or adolescen*)).mp.	Expanded to include mental health conditions
19.	exp Mental Disorders/ or exp Depressive Disorder, Major/ or exp Suicide/ or exp Depression/ or exp Suicidal Ideation/ or exp Depressive Disorder/ or exp Bipolar Disorder/ or exp Suicide, Attempted/ or exp Self-Injurious Behavior/ or exp Substance-Related Disorders/ or exp Social Isolation/	
20.	Limit 19 to female	
21.	((((sex* or viral* or virus* or bacteria*) adj3 diseas*) or HIV or STD* or AIDS) adj3 (female* or wom#n or girl* or child* or adolescen*)).mp.	
22.	exp Sexually Transmitted Diseases, Viral/ or exp Sexually Transmitted Diseases/	
23.	Limit 22 to female	
24	13 or 14 or 15 or 17 or 18 or 20 or 21 or 23	
25.	(AUSTRALIA* or AUSTRIA* or BELGIUM* or belgian* or CANAD* or CHILE* or CZECH* or DENMARK* or danish* or ESTONIA* or FINLAND or finnish or FRANCE or French or GERMAN* or GREECE or greek* or HUNGAR* or ICELAND* or IRELAND or irish or ISRAEL* or ITALY or italian* or JAPAN* or KOREA* or LATVIA* or LUXEMB* or MEXIC* or NETHERLAND* or holland* or dutch or "low countries" or europe* or "NEW ZEALAND*" or NORWAY or	OECD countries

	norwegian* or POLAND or polish or PORTUGAL or portuguese or SLOVAK* or SLOVENIA* or SPAIN or spanish or SWED* or SWITZERLAND or swiss or TURK* or "UNITED KINGDOM" or "UK" or britain or british or england or english or scot* or wales or welsh or UNITED STATES or "USA" or america*).mp.	
26.	randomized controlled trial.pt.	Study types not relevant for qualitative evidence
27.	controlled clinical trial.pt.	
28.	randomized.ab.	
29.	placebo.ab.	
30.	clinical trials as topic.sh.	
31.	randomly.ab.	
32.	trial.ti.	
33.	(animals not (humans and animals)).sh.	
34.	26 or 27 or 28 or 29 or 30 or 31 or 32	
35.	34 not 33	
36.	11 and 12 and 24	
37.	4 and 12	
38.	4 or 36 or 37	
39.	38 and 25	
40.	39 not 35	
41.	40 not 33	
