# Supplementary material for: What are the experiences of seeking, receiving and providing FGM-related healthcare? Perspectives of health professionals and women/girls who have undergone FGM: protocol for a systematic review of qualitative evidence
Source: BMJ Open. 2017 Dec 14;7(12):e018170. doi: 10.1136/bmjopen-2017-018170 (PMC5736050; doi:10.1136/bmjopen-2017-018170)
Supplement: Author's manuscript [file bmjopen-2017-018170.draft_revisions.pdf]

BMJ Open is committed to open peer review. As part of this commitment we make the peer review history of every article we publish publicly available.

When an article is published we post the peer reviewers' comments and the authors' responses online. We also post the versions of the paper that were used during peer review. These are the versions that the peer review comments apply to.

The versions of the paper that follow are the versions that were submitted during the peer review process. They are not the versions of record or the final published versions. They should not be cited or distributed as the published version of this manuscript.

BMJ Open is an open access journal and the full, final, typeset and author-corrected version of record of the manuscript is available on our site with no access controls, subscription charges or pay-per-view fees (http://bmjopen.bmj.com).

If you have any questions on BMJ Open's open peer review process please email editorial.bmjopen@bmj.com

BMJ Open

The experiences of seeking, receiving and providing FGM-related healthcare: perspectives of health professionals and women / girls who have undergone FGM; Protocol for qualitative evidence synthesis

Journal:	*BMJ Open*
Manuscript ID	bmjopen-2017-018170
Article Type:	Protocol
Date Submitted by the Author:	09-Jun-2017
Complete List of Authors:	Evans, Catrin; University of Nottingham School of Health Sciences, School of Health Sciences<br>Tweheyo, Ritah; University of Nottingham School of Health Sciences, School of Health Sciences<br>McGarry, Julie; University of Nottingham,<br>Eldridge, Jeanette; University of Nottingham School of Health Sciences, Research and Learning Services,<br>McCormick, Carol; Queen's Medical Centre<br>Nkoyo, Valentine; Mojatu Foundation<br>Higginbottom, Gina; University of Nottingham School of Health Sciences
**Primary Subject Heading**:	Evidence based practice
Secondary Subject Heading:	Public health, Evidence based practice, Qualitative research, Health services research, Health policy
Keywords:	Female Genital Mutilation/Cutting, qualitative evidence synthesis, immigrant health, health professional perspectives, migrant women and girls, systematic review

SCHOLARONE™
Manuscripts

Title: **The experiences of seeking, receiving and providing FGM-related healthcare: perspectives of health professionals and women / girls who have undergone FGM; Protocol for qualitative evidence synthesis**

Authors:
Dr. Catrin Evans
Associate Professor, and Director of Global Engagement
School of Health Sciences, University of Nottingham, Queens Medical Centre, Nottingham, NG7 2HA. Email: Catrin.Evans@nottingham.ac.uk
orcid.org/0000-0002-5338-2191

Dr. Ritah Tweheyo (**corresponding author**)
Research Fellow, School of Health Sciences, University of Nottingham, Queens Medical Centre, Nottingham, NG7 2HA. Email: Ritah.Tweheyo@nottingham.ac.uk
orcid.org/0000-0002-4609-7253

Dr. Julie McGarry
Associate Professor and Chair of the Domestic Violence and Abuse Integrated Research Group, School of Health Sciences, University of Nottingham, NG7 2HA
orcid.org/0000-0002-7629-2447

Jeanette Eldridge
Senior Research Librarian- Medicine & Health Sciences, Library Research & Learning Services, University of Nottingham, NG7 2RD
orcid.org/0000-0001-7341-4587

Carol McCormick
FGM Specialist Midwife, Nottingham University Hospital Trust

Valentine Nkoyo
Director- Mojatu Foundation, Founder of Nottingham Community FGM Steering Group and FGM Survivors Club

Prof. Gina Marie Awoko Higginbottom MBE,
The Mary Seacole Professor of Ethnicity and Community Health
School of Health Sciences, University of Nottingham Queens Medical Centre, Nottingham, NG7 2HA
orcid.org/0000-0001-8851-0985

Word Count: 3,178 words

For peer review only - http://bmjopen.bmj.com/site/about/guidelines.xhtml

Abstract

Introduction

Female Genital Mutilation (FGM) is an issue of global concern. High levels of migration mean that healthcare systems in higher-income western countries are increasingly being challenged to respond to the care needs of affected communities. Research has identified significant challenges in provision of, and access to, quality FGM-related healthcare. The lack of confidence and competence among health professionals in providing appropriate care calls for urgent need for evidence-based service development in this area. This study will involve two systematic reviews of qualitative evidence to explore the experiences, needs, barriers and facilitators to seeking and providing FGM-related healthcare in high income (OECD) countries, from the perspectives of: (1) women and girls who have undergone FGM and (2) health professionals.

Review methods

Twelve databases including MEDLINE, EMBASE, PsycInfo, ASSIA, Web of Science, ERIC, CINAHL, and POPLINE will be searched. Relevant grey literature will be identified from digital sources and professional networks.

Two reviewers will independently screen, select and critically appraise the studies. Study quality will be assessed using the JBI-QARI appraisal tool. Findings will be extracted and entered into NVivo software. Synthesis by inductive thematic analysis, including in-depth reading, line by line coding of the findings, development of descriptive themes and re-coding to higher level analytical themes. Confidence in the review findings will be assessed using the GRADE-CERQual approach. Findings will be integrated into a comprehensive set of recommendations for policy and practice.

Dissemination

The syntheses will be reported as per the ENTREQ statement. Two reviews will be published in peer-reviewed journals and an integrated report disseminated at stakeholder engagement events.

Registration

The study is registered with the international prospective register of systematic reviews (PROSPERO): CRD42015030001:2015 and CRD42015030004: 2015.

Funder

The National Institute Health Research (NIHR) Health Service Research and Delivery Programme (NIHR 1115/137/04).

The researchers have no conflicts of interest.

For peer review only

For peer review only - http://bmjopen.bmj.com/site/about/guidelines.xhtml

Strengths and Limitations of this Study:

- Will illuminate health professional, organisational and personal factors that influence choices and behaviour on providing FGM-care and following FGM management protocols.
- Will develop an in-depth understanding of the care seeking choices, barriers and experiences across the life course (not just maternity settings).
- Will synthesize research in similar high-income contexts so that findings can be directly translated into interventions and service initiatives across OECD countries that share similar challenges in service provision and uptake.
- This is a participatory collaborative project involving lay representatives from inception through to dissemination.

Key words:
FGM, Female Genital Mutilation/Cutting, systematic review, qualitative evidence synthesis, migrant women and girls, FGM-related healthcare, health professional perspectives, immigrant health, OECD countries.

For peer review only

Introduction

Female Genital Mutilation (FGM) refers to all procedures that involve the partial or total removal of the external female genitalia or other injury to the female genital organs for non-medical reasons [1]. The practice is illegal in most countries and is an internationally recognised violation of the rights of women and girls. FGM is associated with significant negative physical, psychological and sexual health sequelae [2]. In the short term, these include infection, urinary retention or injury to other tissues (e.g. vaginal fistulae). In the longer term, they can include psychological problems, post-traumatic stress disorder, painful intercourse and other sexual problems, relationship problems, chronic pain, chronic infections, infertility and complications in childbirth. It is essential therefore that affected women and girls have access to services that can identify and meet these multiple complex health needs, and that include mental as well as physical healthcare provision [3-5].

There are 30 countries where FGM is traditionally practised, mainly from Africa and parts of Asia [6]. However, increased migration means that FGM is now regularly seen within health services in the UK [7] and other higher income 'receiving' countries where refugees and migrants from practising countries have settled [8]. Hence, health services and health professionals in these countries are increasingly being challenged to develop appropriate services and to care for women/girls who have undergone FGM in a culturally sensitive manner. Migrant's access to healthcare in receiving countries can be problematic however due to a range of socio-economic, legal, language and cultural barriers [9 10]. The ways in which these factors intersect to affect appropriate care delivery for women/girls who have undergone FGM – a particularly sensitive and personal issue - is currently unclear.

Existing evidence on women's and health professionals' experiences around FGM is primarily orientated to maternity care delivery, with much less attention devoted to care delivery in other settings, or for other problems or at other stages of the life course [5 11] . The available evidence from women however highlights a number of concerns, including not knowing about specialist services, feeling unable to talk about FGM, feeling judged and experiencing fear, pain and isolation [12-20]. Likewise, evidence from health professionals indicates that they lack confidence and competence in caring for, and talking about, FGM [21-25]. Interestingly, even where training has been given and where clear care pathways and protocols exist, problems may still remain. For example, a recent study of FGM management in a large London maternity unit found that, in spite of the existence of guidelines and training, clinical care for women/girls with FGM was sub-optimal [26]. The maternity unit had access to a FGM specialist service, but 41% of women with FGM were not identified until

For peer review only - http://bmjopen.bmj.com/site/about/guidelines.xhtml

they arrived in the labour ward. Hence, even though a specialist service existed, it was not being optimally used to benefit women with FGM, and a significant percentage of opportunities were missed to provide women with specialist care. Similar findings were reported from a study in a maternity unit in Switzerland where, in spite of staff training and the existence of clear guidelines, FGM was correctly identified and managed in only 34 (26.4%) of 129 cases reviewed [27]. The reasons for this lack of adherence to protocols are unclear however and there is a need to explore in more depth how organizational and personal factors may influence health professionals' views and behaviour in this area [22 23 25 28-35].

In the UK, a range of policies and protocols around FGM prevention and care have been developed over the last decade. These set out safeguarding procedures to mitigate risk of FGM and recommend a multi-agency approach to service development [36 37]. In the UK, these include; a declaration to end FGM in 2014, the establishment of a National FGM Prevention Programme, development of inter-collegiate guidelines [38-40], mandatory recording using the SCCI2026 FGM Enhanced Dataset Information Standard, the Serious Crime Act 2015, and mandatory reporting of girls at risk of FGM [41]. Many other OECD countries have similar laws barring FGM, including direct prosecution of suspects continuing the practice [37 42]. All EU countries consider FGM a crime of serious bodily harm, with legal measures to safeguard minors, and professional provisions for disclosure and duty to report [37]. Approaches to prosecution are varied, resulting in calls for efforts to improve and correct the inadequate care of women and girls who have undergone FGM [43], specifically for multidisciplinary approaches [11 44]. It is currently unknown how these legal and regulatory provisions impact upon on the experience of seeking and providing healthcare from patients' and health professionals' perspectives.

Much of the existing body of research around FGM relates to understanding the practice of FGM [45], the prevention of FGM [46-48] and the psycho-social and clinical consequences of FGM [2 49-51]. Evidence on the effectiveness of different models of care for FGM is currently lacking [52]. In the absence of high quality evidence on the clinical or cost-effectiveness of different types of service provision, decision making around FGM services must be informed by other forms of evidence [53]. Hence, this project proposes to bring together existing qualitative evidence on lay and professional experiences of, and perspectives on, FGM care, and to relate this to service development in higher income (receiving) countries. In doing so, it will provide a vital resource for evidence-informed FGM-related service development and training. Qualitative research can provide insights into factors influencing service acceptability, appropriateness and meaningfulness, and on micro and macro level

For peer review only - http://bmjopen.bmj.com/site/about/guidelines.xhtml

organizational and contextual issues that influence service use and delivery [54-56]. Therefore, the proposed qualitative evidence syntheses around FGM will generate rich data and deep understanding with which to inform new service initiatives and the content and structure of staff training resources [57 58].

There are a number of systematic reviews related to FGM healthcare that have been published to date, but all have taken a multi-context (or 'lumping') approach to the evidence [11 52 59 60] having included research from high and low-income settings across the world. Many key themes from these reviews therefore are drawn from evidence from very different contexts and are not easily transferable to a high-income setting. These countries generally have strong, well-resourced public health systems and FGM is primarily found within their migrant populations. FGM care and its challenges in these settings, will therefore, be linked to other challenges around providing care for migrant populations (such as lack of familiarity with cultural norms, systems or communication issues). This is a very different situation compared with countries where health systems are weak, where FGM is more prevalent, where health providers may have greater exposure to, and indeed, may even be complicit in, FGM. In order to address this shortcoming, the proposed systematic reviews of women's and health professionals' experiences will focus exclusively on evidence from high income OECD contexts.

Aim

The aim of this review will be two-fold: to understand the experiences, needs, barriers and facilitators around seeking and providing FGM-related care from the perspectives of i) women and girls who have undergone FGM, and ii) health professionals. Hence, this will involve 2 reviews of qualitative evidence:

Review 1: ***To explore the experiences of FGM-related healthcare across the life course for women and girls who have undergone FGM.***

Objectives:

From the perspective of women and girls who have undergone FGM: -

i. to illuminate factors that influence FGM-related healthcare seeking and access to health services across the lifecourse,
ii. to explore how quality of care is perceived and experienced in different healthcare settings and with different groups of healthcare professionals,

For peer review only - http://bmjopen.bmj.com/site/about/guidelines.xhtml

iii. to characterise and explain elements of service provision considered important for the provision of acceptable and appropriate healthcare,
iv. to describe factors perceived to influence open discussion and communication around FGM (including prevention) with health professionals.

Review 2: *To explore the views on, and experiences of, all cadres of health professionals in providing care for women/girls who have undergone FGM.*

Objectives:

From the perspective of health professionals:

i. to explore how quality of care for women/girls who have undergone FGM is perceived in different healthcare settings and amongst different professional groups,
ii. to characterise and explain elements of service provision considered important for the provision of high quality care to women/girls who have undergone FGM,
iii. to illuminate factors perceived to facilitate or hinder appropriate provision of care for women and girls who have undergone FGM,
iv. to identify processes and practices perceived to influence open discussion and communication around FGM (including prevention) with women/girls from affected communities.

Methodology and Methods

The reviews' aims and objectives were generated together with community organisations working in the field of FGM who continue to be involved as co-investigators and advisors. Their perspectives will be integrated into project activities at every stage. The reviews seek to identify insights about lay/health professional experiences of FGM-related healthcare and perceived appropriateness and acceptability of services. These are questions best answered by qualitative research [54 55] – hence the specific focus on qualitative evidence.

There are many possible approaches to qualitative evidence synthesis, with most discussions in this area characterizing the different types along a continuum between aggregation and interpretation [61]. Where the purpose of a synthesis is to generate new theoretical insights, a highly interpretive approach such as meta-ethnography may be most suitable, informed by an idealist epistemological stance. However, where the purpose is to inform policy or practice a more aggregative or thematic approach informed by a realist

For peer review only - http://bmjopen.bmj.com/site/about/guidelines.xhtml

epistemology is often advocated [62]. The latter is also suggested in cases where the existing evidence is likely to be descriptive (as in much health services research) rather than highly theoretical or conceptual [57]. An initial scoping of the literature suggests that this is the case for the proposed syntheses. A thematic synthesis approach involves using thematic analysis techniques to identify key concepts/themes within primary research studies [63 64]. Synthesis involves an iterative and inductive process of grouping themes into overarching categories and exploring the similarities, differences and relationships between them. Thematic synthesis explicitly aims to move beyond generating a list of descriptive themes (as would be the case in meta-aggregation) in order to identify new, higher order, analytical insights that can contribute to new understandings of a phenomenon. Review recommendations however are clearly formulated to inform policy and practice. As such, it is considered the most suitable approach for the two proposed systematic reviews [65].

The findings of the two reviews will be reported separately and then, where appropriate, integrated to enable greater understanding of key issues or concepts when presented from multiple perspectives. The findings will be brought together into a comprehensive set of recommendations for service development, community engagement and health professional education.

Figure 1: Evidence syntheses to improve FGM-related care

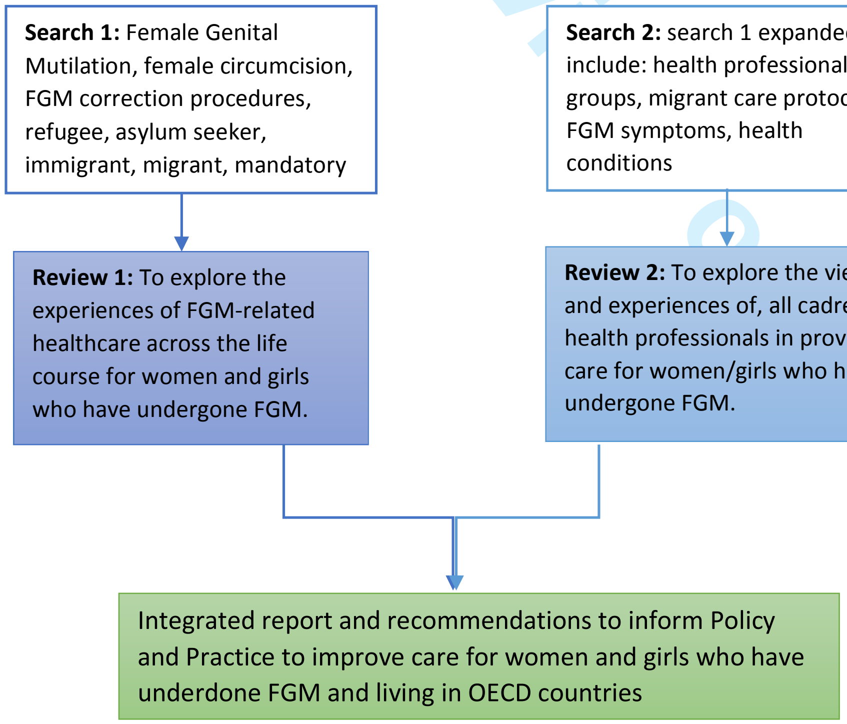

For peer review

Approach to searching

The project will implement a comprehensive search strategy to gather all available and accessible studies, including peer reviewed articles and grey literature. Primary research articles in the form of journal papers, research/evaluation reports, theses and dissertations will be collected. Reference lists from primary papers and key reviews will be hand searched to identify additional papers. There will be no language or date limits employed when searching.

Data Sources

Searching will be done in at least 12 databases to include Medline (Ovid), Embase, Scopus, PsycINFO, Web of Science, ASSIA, CINAHL, ERIC, MIDIRS. Additional searches will be carried out in relevant indexes such as POPLINE, grey literature databases including: British library, NICE evidence services, index to theses, Networked Digital Library of Theses and Dissertations (NDLTD), ProQuest and other accessible digital thesis and dissertation repositories.

Search Strategy

The search strategy was developed from an initial scoping search undertaken to establish relevant search terms and potential databases. The Librarian (JE) together with RT will develop further and conduct all the searches. The comprehensive search will be tailored to each of the listed databases for both peer reviewed and grey literature and results reported in a flowchart following PRISMA guidance. Electronic searches are summarised in Fig 2 and an initial detailed search strategy is attached in Appendix 1.

For peer review only

For peer review only - http://bmjopen.bmj.com/site/about/guidelines.xhtml

Figure 2: *Search Strategy flowchart*

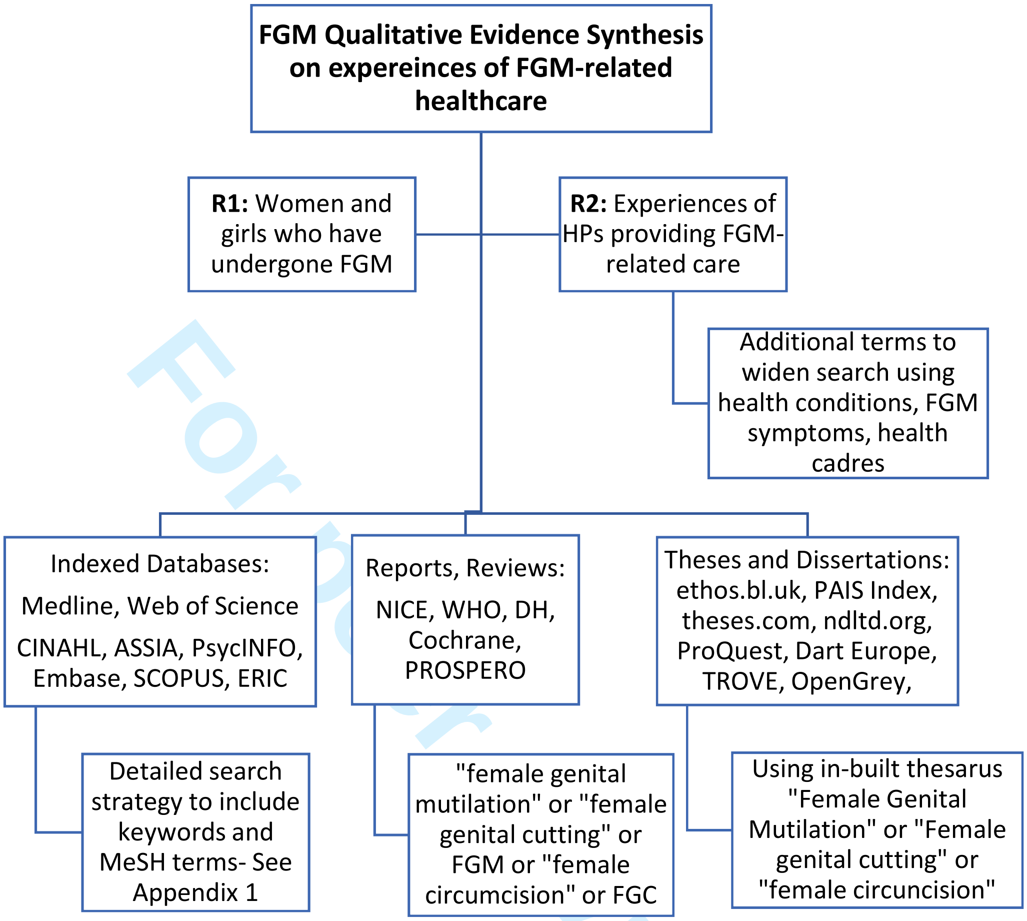

Screening and Eligibility

All returned results from the searches will be entered into the EndNote reference manager programme. Screening will be undertaken independently by 2 reviewers using title and abstract, and studies selected if they meet the a-priori selection criteria (see table 1). In case of disagreement, there will be recourse to a 3rd reviewer when required.

The inclusion criteria are:

- **Population:** Women and girls who have undergone FGM, and their experience of FGM-related healthcare, or healthcare professionals or students involved in the care of women and girls that have undergone FGM.
- **Phenomenon of Interest:** Experiences of seeking, receiving or providing FGM-related healthcare across the life course.

For peer review only - http://bmjopen.bmj.com/site/about/guidelines.xhtml

- **Context or Setting:** Studies conducted in OECD/high-income countries as described by the World Bank (List attached in Appendix 1).
- **Study type:** Any type of qualitative study and any type of mixed methods study that reports qualitative findings.
- **Search Limits:** No language or date limits. The abstracts of articles not in English will be translated and assessed. Those that meet the screening criteria will then be professionally translated into English for appraisal and data extraction.
- **Exclusion:** Any papers or research that was not undertaken on women and girls who have undergone FGM, or healthcare professionals, or not in OECD countries. Studies will be excluded if they do not describe FGM-related experiences of healthcare or provision of healthcare.

Table 1: *Screening Checklist*

**Screening Criteria**	**Y**	**N**	**U**
Does the study report findings related to……………..<br>• Experiences of seeking and receiving **healthcare** related to FGM?<br>• Experiences of providing **healthcare** to women/girls who have undergone FGM?			
Is the study conducted in an OECD country?			
Is it a qualitative study?<br>Or<br>Does it report qualitative findings from a mixed methods study?			
Are the participants………………..<br>• Women or girls who have undergone FGM?<br>• Health professionals?			

Study Appraisal of Quality:

The role of critical appraisal in qualitative evidence synthesis is contested and there is lack of agreement over: (i) the appropriateness of excluding studies, (ii) the potential impact (or not) of excluding eligible papers on review outcomes, and, (iii) the criteria on which quality should be established [66-69]. For these reasons, the project will adopt an inclusive approach to critical appraisal, using the appraisal process to enable an in-depth understanding of each paper and to facilitate a critical, questioning approach to the study findings [70]. Studies will not be excluded on the basis of quality, rather, the quality assessment will be used: (i) to judge the relative contribution of each study to the overall synthesis, and, (ii) to assess the

For peer review only - http://bmjopen.bmj.com/site/about/guidelines.xhtml

methodological rigour of each study as part of a process of assessing confidence in the review findings [71]. The quality of included studies will be assessed independently by 2 reviewers using the Joanna Briggs Institute Qualitative Assessment and Review Instrument (JBI-QARI) [72 73]. This tool has been found to be more coherent and more sensitive to assessment of validity than other commonly used tools [74]. An extract summary of the appraisal items is listed in Table 2.

For peer review only

For peer review only - http://bmjopen.bmj.com/site/about/guidelines.xhtml

***Table 2**: Extracted JBI Critical Appraisal Checklist*

Extracted JBI Qaulitative Assessement and Review Instrument (QARI)															
**Record details (Full reference)**	Is there congruity between the stated philosophical perspective and the research methodology?	Is there congruity between the research methodology and the research question or objectives?	Is there congruity between the research methodology and the methods used to collect data?	Is there congruity between the research methodology and the representation and analysis of data?	Is there congruity between the research methodology and the interpretation of results?	Is there a statement locating the researcher culturally or theoretically?	Is the influence of the researcher on the research, and vice- versa, addressed?	Are participants, and their voices, adequately represented?	Is the research ethical according to current criteria or, for recent studies, and is there evidence of ethical approval by an appropriate body?	Do the conclusions drawn in the research report flow from the analysis, or interpretation, of the data?	**Score**	**Include**	**Exclude**	**Seek further info**	**Comments (Including reason for exclusion)**
	Q1	Q2	Q3	Q4	Q5	Q6	Q7	Q8	Q9	Q10	/10				

***Table 3**: Data Extraction tool to include all the results and findings sections of each included study*

Data extraction tool- modified JBI tool for FGM Systematic Review												
Study (Name and Authors)	Methodology	Methods	Purpose/ Phenomena of interest	Setting	Geographical	Cultural	Participants	Data analysis	Findings/ Results	Authors Conclusion	Reviewers Comments	Outcomes

For peer review only - http://bmjopen.bmj.com/site/about/guidelines.xhtml

Data extraction

Data extraction will primarily be undertaken by one reviewer with a 2nd and 3rd reviewer each checking a random sample of the articles for completeness in extraction. Data will be extracted using a modified JBI data extraction form (Table 3), to include details of the phenomenon of interest, population, context, study methodology, methods, and findings of significance to the review objectives [72 75]. If studies lack relevant detail, authors will be contacted to provide further information. The 'results/findings' sections from each study will be entered into NVivo for further analysis and synthesis.

Synthesis

Analysis will be conducted by 2 reviewers in consultation with the research team. We shall carry out an inductive-thematic analysis for each review [57], conducted in 4 phases: (i) in-depth reading and immersion in the data, (ii) coding of findings that are connected to FGM-related healthcare, (iii) re-coding to higher level themes and (iv) interpretive synthesis. If appropriate, the latter stage may involve the development of models or frameworks to identify and display relationships between, and patterns within, the analytical themes. Where possible, the analytical themes will be formulated as 'directive' findings indicating clear messages and/or suggesting clear lines of action for policy and practice [72].

Assessment of Confidence

The review findings will be assessed using the GRADE-CERQUAL guidelines [71]. The assessment of confidence in the evidence for an individual review finding considers four elements: (i) methodological limitations (the extent to which there are problems in the design or conduct of primary studies that contributed to evidence of a review finding); (ii) relevance (the extent to which the body of evidence from the primary studies supporting a review finding is applicable to the context specified in the review question); (iii) coherence (whether the finding is well grounded in data from the primary studies and can provide a convincing explanation for pattern found in the data); and (iv) adequacy of data (an overall determination of the degree of richness and quantity of data supporting a review finding) [71:2-4]. Based on the assessment, each review finding will be assigned one of four levels of confidence: high, moderate, low and very low.

Presenting and reporting the review

Each review will be reported in accordance with the 'Enhancing Transparency in Reporting the Synthesis of Qualitative Research' (ENTREQ) statement [76], appropriate for qualitative evidence synthesis, which consists of 21 items- see Table 4.

For peer review only - http://bmjopen.bmj.com/site/about/guidelines.xhtml

Table 4: *Summary of the 21 items from the ENTREQ Statement*

No	Item
1	Aim
2	Synthesis methodology
3	Approach to searching
4	Inclusion criteria
5	Data sources
6	Electronic Search strategy
7	Study screening methods
8	Study characteristics
9	Study selection results
10	Rationale for appraisal
11	Appraisal items
12	Appraisal process
13	Appraisal results
14	Data extraction
15	Software
16	Number of reviewers
17	Coding
18	Study comparison
19	Derivation of themes
20	Quotations
21	Synthesis output

Dissemination

The 2 qualitative systematic reviews will be published separately in peer reviewed journals and an integrated report compiled and widely disseminated to UK stakeholders through stakeholder engagement events, round-table discussions with different stakeholder groups, and tailored materials for each of the stakeholder groups (academic, health professionals, policy makers/commissioners, FGM communities and community organisations.

Conclusion

The participatory collaborative approach to this qualitative evidence synthesis project will enhance the multidisciplinary interpretation of findings in order to inform stakeholders and the development of FGM-care initiatives for improved confidence and quality of service provision to women and girls who have undergone FGM.

Conflict of interest

The authors declare no competing interests in the development of this manuscript.

For peer review only

Acknowledgements

This systematic review is funded under Project: ***Improving Care for Women and Girls who have undergone Female Genital Mutilation (FGM): Qualitative Evidence Synthesis***. Funded by the National Institute Health Research (NIHR) Health Service Research and Delivery Programme (NIHR 1115/137/04).

Author Contributions

CE conceptualised the project and prepared the project documents as PI in collaboration with the team members GH, JM, CC, and VN.

JE developed the search strategy and will carry out the searches

RT prepared the manuscript with input from team members.

All team members reviewed and approved this manuscript.

References

1. WHO. Female genital mutilation: fact sheet. Geneva: World Health Organisation 2014.
2. WHO. A Systematic Review of the Health Complications of Female Genital Mutilation including Sequelae in Childbirth. Geneva: WHO, 2000.
3. HM Government. Multi-agency practice guidelines: Female genital mutilation. London, 2014.
4. Creighton S, Hodes D. Female genital mutilation (FGM): Chapter 3: Annual Report of the Chief Medical Officer: The Health of the 51%: Women. In: Department of Health, ed. London: Department of Health, 2015.
5. WHO. WHO guidelines on the management of health complications from female genital mutilation. Geneva: WHO, 2016.
6. United Nations Children's Fund. Female Genital Mutilation/Cutting: A Global Concern. New York: UNICEF, 2016.
7. Macfarlane A, Dorkenoo E. Female Genital Mutilation in England and Wales: Updated statistical estimates of the numbers of affected women living in England and Wales and girls at risk Interim report on provisional estimates. 2014
8. Van Baelen L, Ortensi L, Leye E. Estimates of first-generation women and girls with female genital mutilation in the European Union, Norway and Switzerland. *The European journal of contraception & reproductive health care : the official journal of the European Society of Contraception* 2016;21(6):474-82.
9. Sarría-Santamera A, Hijas-Gómez AI, Carmona R, et al. A systematic review of the use of health services by immigrants and native populations. *Public Health Reviews* 2016;37(1) doi: 10.1186/s40985-016-0042-3
10. Suphanchaimat R, Kantamaturapoj K, Putthasri W, et al. Challenges in the provision of healthcare services for migrants: a systematic review through providers' lens. *BMC Health Serv Res* 2015;15:390. doi: 10.1186/s12913-015-1065-z
11. Zurynski Y, Sureshkumar P, Phu A, et al. Female genital mutilation and cutting: a systematic literature review of health professionals' knowledge, attitudes and clinical practice. *BMC international health and human rights* 2015;15:32.

For peer review only - http://bmjopen.bmj.com/site/about/guidelines.xhtml

12. Berggren V, Bergstrom S, Edberg AK. Being different and vulnerable: Experiences of immigrant African women who have been circumcised and sought maternity care in Sweden. *Journal of Transcultural Nursing* 2006;17(1):50-57.
13. Chalmers B, Hashi KO. 432 Somali women's birth experiences in Canada after earlier female genital mutilation. *Birth* 2000;27(4):227-34.
14. Moxey JM, Jones LL. A qualitative study exploring how Somali women exposed to female genital mutilation experience and perceive antenatal and intrapartum care in England. *BMJ Open* 2016;6 (1) (no pagination)(e009846)
15. Vloeberghs E, Van der kwaak A, Knipscheer J, et al. Coping and chronic psychosocial consequences of female genital mutilation in the Netherlands. *Ethnicity & Health* 2012;17(6):677-95.
16. d'Entremont M, Smythe L, McAra-Couper J. The sounds of silence: A hermeneutic interpretation of childbirth post excision. *Health Care for Women International* 2014;35(3):300-19. doi: http://dx.doi.org/10.1080/07399332.2013.838245
17. Hussein E. Women's experiences, perceptions and attitudes of female genital mutilation: the Bristol PEER study: Foundation for Women's Health Research and Development (FORWARD), 2010.
18. Upvall MJ, Mohammed K, Dodge PD. Perspectives of Somali Bantu refugee women living with circumcision in the United States: a focus group approach. *Int J Nurs Stud* 2009;46(3):360-8. doi: 10.1016/j.ijnurstu.2008.04.009
19. Vangen S, Johansen REB, Sundby J, et al. Qualitative study of perinatal care experiences among Somali women and local health care professionals in Norway. *Eur J Obstet Gynecol Reprod Biol* 2004;112(1):29-35. doi: 10.1016/s0301-2115(03)00313-0
20. Recchia N, McGarry J. "Don't judge me": narratives of living with FGM. *Ethnicity and Inequalities in Health and Social Care* 2017;10(1):4-13.
21. Clayton-Hathway K. A pilot evaluation of health services for communities affected by FGM in Oxfordshire: Final report. Oxford: Oxford Against Cutting, 2016.
22. Dawson AJ, Turkmani S, Varol N, et al. Midwives' experiences of caring for women with female genital mutilation: Insights and ways forward for practice in Australia. *Women and Birth* 2015;28(3):207-14. doi: http://dx.doi.org/10.1016/j.wombi.2015.01.007
23. Degni F, Suominen S, Essén B, et al. Communication and Cultural Issues in Providing Reproductive Health Care to Immigrant Women: Health Care Providers' Experiences in Meeting Somali Women Living in Finland...[corrected] [published erratum appears in J IMMIGRANT MINORITY HEALTH 2012; 04(2):344]. *Journal of Immigrant & Minority Health* 2012;14(2):330-43 14p. doi: 10.1007/s10903-011-9465-6
24. Johansen E. Care for infibulated women giving birth in Norway. *Medical Anthropology Quarterly* 2006;20(4):516-44.
25. Kaplan-Marcusan A, Toran-Monserrat P, Moreno-Navarro J, et al. Perception of primary health professionals about female genital mutilation: from healthcare to intercultural competence. *BMC health services research* 2009;9:11. doi: 10.1186/1472-6963-9-11
26. Zenner N, Liao LM, Richens Y, et al. Quality of obstetric and midwifery care for pregnant women who have undergone female genital mutilation. *J Obstet Gynaecol* 2013;33(5):459-62. doi: 10.3109/01443615.2013.767785
27. Abdulcadir J, Dugerdil A, Boulvain M, et al. Missed opportunities for diagnosis of female genital mutilation. *International Journal of Gynaecology and Obstetrics* 2014;125(3):256-60. doi: 10.1016/j.ijgo.2013.11.016
28. Cappon S, L'Ecluse C, Clays E, et al. Female genital mutilation: Knowledge, attitude and practices of Flemish midwives. *Midwifery* 2015;31(3):e29-35 1p. doi: 10.1016/j.midw.2014.11.012
29. Fawcett L. Somali refugee women and their U.S. healthcare providers: Knowledge, perceptions and experiences of childbearing. *Dissertation Abstracts International Section A: Humanities and Social Sciences* 2015;75(8-A(E)):No Pagination Specified.

For peer review only - http://bmjopen.bmj.com/site/about/guidelines.xhtml

30. Isman E, Mahmoud Warsame A, Johansson A, et al. Midwives' Experiences in Providing Care and Counselling to Women with Female Genital Mutilation (FGM) Related Problems. *Obstetrics and gynecology international* 2013;2013:785148. doi: 10.1155/2013/785148
31. Kaplan-Marcusan A, Del Rio NF, Moreno-Navarro J, et al. Female genital mutilation: perceptions of healthcare professionals and the perspective of the migrant families. *BMC Public Health* 2010;10:193. doi: 10.1186/1471-2458-10-193
32. León-Larios F, Casado-Mejía R. Knowledge, attitudes and professional experiences of midewives in primary health care in Seville on female genital mutilation. *Matronas Profesion* 2014;15(2):56-61 6p.
33. Tamaddon L, Johnsdotter S, Liljestrand J, et al. Swedish health care providers' experience and knowledge of female genital cutting. *Health Care Women Int* 2006;27(8):709-22. doi: http://dx.doi.org/10.1080/07399330600817741
34. Widmark C, Leval A, Tishelman C, et al. Obstetric care at the intersection of science and culture: Swedish doctors' perspectives on obstetric care of women who have undergone female genital cutting. *Journal of Obstetrics and Gynaecology* 2010;30(6):553-58.
35. Widmark C, Tishelman C, Ahlberg BM. A study of Swedish midwives' encounters with infibulated African women in Sweden. *Midwifery* 2002;18(2):113-25.
36. HM Government. Multi-agency statutory guidance on female genital mutilation, https://www.gov.uk/government/uploads/system/uploads/attachment_data/file/512906/Multi_Agency_Statutory_Guidance_on_FGM__-_FINAL.pdf2016 [accessed Accessed 17/05/17.
37. Leye E, Deblonde J, García-Añón J, et al. An analysis of the implementation of laws with regard to female genital mutilation in Europe. *Crime, law and social change* 2007;47(1):1-31. doi: http://dx.doi.org/10.1007/s10611-007-9055-7
38. Royal College of Midwives, Royal College of Nursing, Royal College of Obstetricians and Gynaecologists, et al. Intercollegiate guidelines tackling FGM in the UK: Intercollegiate recommendations for identifying, recording and reporting. London, 2013.
39. Royal College of Nursing. Female genital mutilation: An RCN resource for nursing and midwifery practice (2nd ed). London: RCN, 2015.
40. Royal College of Obstetricians and Gynaecologists. Green-top guideline no.53: Female genital mutilation and its management. London, 2015.
41. Department of Health. Female Genital Mutilation Risk and Safeguarding; Guidance for professionals. London: Crown copyright, 2016.
42. United States. Agency for International D. Female genital mutilation / cutting: United States Government's response: [Washington, D.C.], USAID, 2014 Jul 21., 2014:[3] p.
43. Rouzi AA, Alturki F. Female genital mutilation/cutting: an update. *Clinical and experimental obstetrics & gynecology* 2015;42(3):300-3.
44. Creighton SM, Dear J, de Campos C, et al. Multidisciplinary approach to the management of children with female genital mutilation (FGM) or suspected FGM: service description and case series. *BMJ open* 2016;6(2):e010311.
45. Berg RC, Denison E. A Tradition in Transition: Factors Perpetuating and Hindering the Continuance of Female Genital Mutilation/Cutting (FGM/C) Summarized in a Systematic Review. *Health Care for Women International* 2013;34(10):837-59.
46. Varol N, Turkmani S, Black K, et al. The role of men in abandonment of female genital mutilation: a systematic review. *BMC Public Health* 2015;15(1):1034. doi: 10.1186/s12889-015-2373-2
47. Norwegian Knowledge Centre for the Health Services. Effectiveness of interventions designed to reduce the prevalence of female genital mutilation-cutting. Oslo, 2009.
48. Berg RC, Denison EM. A realist synthesis of controlled studies to determine the effectiveness of interventions to prevent genital cutting of girls. *Paediatrics and international child health* 2013;33(4):322-33. doi: 10.1179/2046905513Y.0000000086

For peer review only

For peer review only - http://bmjopen.bmj.com/site/about/guidelines.xhtml

49. Mulango P, Hollins-Martin C, McAndrew S. The psychological impact of Female Genital Mutilation-Cutting 'FGM-C' on girls-women's mental health a narrative literature review. *Journal of Reproductive and Infant Psychology* 2014;32(5) doi: 10.1080/02646838.2014.949641
50. Norwegian Knowledge Centre for the Health Services. Psychological, social and sexual consequences of female genital mutilationcutting-FGMC-A systematic review of quantitative studies. Oslo, 2010.
51. Berg RC, Underland V. The obstetric consequences of female genital mutilation/cutting: a systematic review and meta-analysis. *Obstetrics & Gynecology International* 2013;2013:496564. doi: 10.1155/2013/496564
52. Sunday-Adeoye I, Serour G. Management of health outcomes of female genital mutilation: Systematic reviews and evidence syntheses. *International journal of gynaecology and obstetrics: the official organ of the International Federation of Gynaecology and Obstetrics* 2017;136 Suppl 1:1-2.
53. Abdulcadir J, Rodriguez MI, Say L. Research gaps in the care of women with female genital mutilation: An analysis. *British Journal of Obstetrics and Gynaecology* 2015;122(3):294-303. doi: 10.1111/1471-0528.13217
54. Popay J, Williams G. Qualitative research and evidence based healthcare. *Journal of the Royal Society of Medicine* 1998;35(91):32-37.
55. Pope C, Mays N. Reaching the parts other methods cannot reach: an introduction to qualitative methods in health and health services research. *British Medical Journal* 1995(6996):42-45.
56. Medical Research Council. Developing and evaluating complex interventions: new guidance. London 2008.
57. Thomas J, Harden A. Methods for the thematic synthesis of qualitative research in systematic reviews. *BMC Medical Research Methodology* 2009;8:45.
58. Ring N, Ritchie K, Mandava L, et al. A guide to synthesising qualitative research for researchers undertaking health technology assessment and systematic reviews. Edinburgh: NHS Quality Improvement Scotland and University of Stirling, 2011.
59. Hannes K, Harden A. Multi-context versus context-specific qualitative evidence syntheses: combining the best of both. *Research Synthesis Methods* 2011;2(4):271-78. doi: 10.1002/jrsm.55
60. Weir MC, Grimshaw JM, Mayhew A, et al. Decisions about lumping vs. splitting of the scope of systematic reviews of complex interventions are not well justified: a case study in systematic reviews of health care professional reminders. *J Clin Epidemiol* 2012;65(7):756-63. doi: 10.1016/j.jclinepi.2011.12.012 [published Online First: 2012/04/14]
61. Barnett-Page E, Thomas J. Methods for the synthesis of qualitative research: a critical review. *BMC Mecial Research Methodology* 2009;9:59.
62. Paterson B. "It looks great but how do I know it fits?": An introduction to meta-synthesis research. In: Hannes H, Lockwood C, eds. Synthesizing Qualitative Research: Choosing the Right Approach. Oxford: Wiley Blackwell BMJ Books 2012:1-21.
63. Boyatzis R. Transforming qualitative data: thematic analysis and code development. London: Sage 1998.
64. Braun V, Clarke V. Using thematic analysis in psychology. *Qualitative Research in Psychology* 2006;3(2):77-101.
65. Noyes J, Booth A, Hannes K, et al. Supplementary guidance for inclusion of qualitative research in Cochrane systematic reviews of interventions, Version 1: Cochrane Collaboration Qualitative Methods Group 2011.
66. Carroll C, Booth A, Lloyd-Jones M. Should We Exclude Inadequately Reported Studies From Qualitative Systematic Reviews? An Evaluation of Sensitivity Analyses in Two Case Study Reviews. *Qualitative Health Research* 2012;22(10):1425-34.

For peer review only - http://bmjopen.bmj.com/site/about/guidelines.xhtml

67. Cohen D, Crabtree B. Evaluative criteria for qualitative research in health care: controversies and recommendations. *Annals of Family Medicine* 2008;6:4.
68. Hannes K, Lockwood C, Pearson A. A comparative analysis of three online appraisal instruments' ability to assess validity in qualitative research. *Qualitative Health Research* 2010;20(12):1736-43. doi: 10.1177/1049732310378656 [published Online First: 2010/07/31]
69. Toye F, Seers K, Allcock N, et al. 'Trying to pin down jelly'-exploring intuitive processes in quality assessment for meta-ethnography. *BMC Medical Research Methodology* 2013;13(1):46.
70. Hannes K, Pearson A. Obstacles to the Implementation of Evidence Based Practice in Belgium: a Worked Example of Meta-Aggregation. In: Hannes K, Lockwood C, eds. Synthesizing Qualitative Research: Choosing the Right Approach: BMJ Books, Wiley Blackwell 2012:21-41.
71. Lewin S, Glenton C, Munthe-Kaas H, et al. Using qualitative evidence in decision making for health and social interventions: An approach to assess confidence in findings from qualitative evidence syntheses (GRADE-CERQUAL). *Plos Medicine* 2015;12(10):e1001895.
72. Pearson A, Robertson-Malt S, Rittenmeyer L. Synthesizing qualitative evidence. Philadelphia: Lippincott-Joanna Briggs Institute 2011.
73. Joanna Briggs Institute (JBI QARI Critical Appraisal Checklist for Interpretive and Critical Research). [Available from: http://joannabriggs.org/assets/docs/jbc/operations/criticalAppraisalForms/JBC_Form_CritAp_IntCrit.pdf.
74. Hannes K, Lockwood C, Pearson A. A comparative analysis of three online appraisal instruments' ability to assess validity in qualitative research. *Qualitative Health Research* 2010;20(12):1739-43.
75. Joanna Briggs Institute (JBI QARI Data Extraction Form for Interpretive and Critical Research). [Available from: http://joannabriggs.org/assets/docs/jbc/operations/dataExtractionForms/JBC_Form_DataE_IntCrit.pdf.
76. Tong A, Flemming K, McInnes E, et al. Enhancing transparency in reporting the synthesis of qualitative research: ENTREQ. *BMC Medical Research Methodology* 2012;12(1):181. doi: 10.1186/1471-2288-12-181

For peer review only

For peer review only - http://bmjopen.bmj.com/site/about/guidelines.xhtml

Revised Appendix 1: Revised Search Strategy

Key databases: Medline, Embase, PsycINFO, CINAHL, POPLINE, ASSIA, Web of Science

MeSH terms
exp Circumcision, Female/
exp Genitalia, Female/ and exp Medicine, Traditional/

Sample Search: Medline: Epub Ahead of Print, In-Process & Other Non-Indexed Citations, Ovid MEDLINE(R) Daily and Ovid MEDLINE(R) 1946 to Present

07/07/16

			Notes
1	exp Circumcision, Female/ or (exp Genitalia, Female/ and exp Medicine, Traditional/)	1141	
2	("female genital mutilation" or "female circumcision" or "female genital cutting" or "traditional female genital surgery" or ((ritual* or traditional* or ceremon* or sociali#ation*) adj3 (practic* or cut or cutting or surg*) adj3 (female* or wom#n or girl* or child* or adolescen*)) or ((circumcis* or excis*) adj3 (female* or wom#n or girl* or child* or adolescen*)) or infibulat* or reinfibulat* or defibulat* or clitoridect*).mp.	2930	Expanded strategy to include "children"<br><br>Original version retrieved 2239 hits
3	(FGM or TFGS).mp.	661	Abbreviations have different meanings in other contexts
4	limit 3 to female	322	…so are limited to female to exclude irrelevant hits
5	1 or 2 or 4	3136	Previous version retrieved 2447 hits – increase due to broadening search and added records since search last run on 11/12/15
6	exp Pregnancy Complications/ or exp Pregnancy High Risk/ or ((poor* or adverse* or complicat* or difficult* or risk*) adj3 (pregnan* or birth* or matern*)).ti,ab.	403648	
7	"Emigrants and Immigrants"/ or Refugees/ or "Transients and Migrants"/ or "Emigration and Immigration"/	42513	
8	(immigrant* or immigrat* or emigrant* or emigrat* or emigre* or expat* or (ex adj pat*) or transients or newcomer* or (new adj comer*) or alien* or incomer* or (in adj comer*)).mp.	75833	

For peer review only - http://bmjopen.bmj.com/site/about/guidelines.xhtml

9	(refugee* or (asylum adj seek*) or asylee* or (displaced adj person*) or exile* or (new adj arrival) or (country adj2 (birth or origin))).mp.	14708	Terms added to cover “refugee, asylum seeker, immigrant, migrant
10	(foreigner* or (foreign adj (born or citizen* or national* or origin*)) or (non adj (citizen* or native*)) or ((adoptive or naturali#ed) adj (citizen* or resident*))).mp.	9144	
11	7 or 8 or 9 or 10	92578	
12	Ethnic Groups/ or (ethnic* or ethno*).mp.	161783	Terms added to cover general and specific ethnic groups who are known to practice FGM (taken from **Draft** Multi-Agency Statutory Guidance on Female Genital Mutilation (for consultation), UK Home Office; issued as Multi-agency statutory guidance on female genital mutilation, 1 April 2016)
13	continental population groups/	18256	
14	exp european continental ancestry group/	58569	
15	exp african continental ancestry group/	78075	
16	exp asian continental ancestry group/	54673	
17	(africa* or "middle east*" or asia* or iran* or iraq* or israel* or oman* or united arab emirat* or UAE or palestin* or india* or indonesia* or malaysia* or pakistan*).mp.	669568	
18	(egypt* or yemen* or sudan* or ethiopi* or somali* or djibouti* or eritrea* or kenya* or uganda* or tanzania* or camero#n* or chad* or niger* or benin* or togo* or ghana* or burkin* or ivory coast* or cote d'ivoire or liberia* or guinea* or sierra leone or leonean* or gambia* or senegal* or mauritan* or mali or malian*).mp.	322529	
19	12 or 13 or 14 or 15 or 16 or 17 or 18	1066509	
20	6 and (11 or 19)	26967	Pregnancy issues AND (migrants OR ethnic groups)
21	exp Mandatory Reporting/	2801	Terms added to cover mandatory reporting, prevention
22	(prevent* or interven* or safeguard* or "duty of care").ti,ab.	1737061	
23	21 or 22	1739400	
24	exp Community Health Workers/ or exp Volunteers/	19172	Terms added to cover health workers, midwives, general practitioners, nurses, support workers, social workers, volunteers, police (= Occupational Groups)
25	exp Occupational Groups/	495573	
26	exp Health Personnel/	424103	
27	exp Midwifery/	16608	
28	exp General Practitioners/ or exp Physicians/	109231	

For peer review only - http://bmjopen.bmj.com/site/about/guidelines.xhtml

29	exp Nurses/ or exp Nurses, Community Health/	76701	
30	exp Nurses' Aides/ or exp Nursing Staff, Hospital/ or exp Nursing Staff/	62619	
31	exp Social Workers/	50	
32	(female* or wom#n or girl* or child* or adolescen*).mp.	8776379	
33	24 or 25 or 26 or 27 or 28 or 29 or 30 or 31	521707	
34	20 and 23 and 33	282	Pregnancy issues AND (migrants OR ethnic groups) AND reporting AND health workers
35	(11 or 19) and 23 and 32 and 33	4393	Female AND (migrants OR ethnic groups) AND reporting AND health workers
36	5 or 34 or 35	7522	FGM OR (Pregnancy issues AND (migrants OR ethnic groups) AND reporting AND health workers) OR Female AND (migrants OR ethnic groups) AND reporting AND health workers

Conference Papers Index: http://search.proquest.com/cpi?accountid=8018 (via ProQuest)

Clinical Trials Registers (for process evaluations)
- www.clinicaltrials.gov
- http://www.controlled-trials.com
- http://www.nhs.uk/Conditions/Clinical-trials/Pages/Registeringresults.aspx
- http://www.isrctn.com/

Theses
- Index to Theses: http://www.theses.com/
- Networked Digital Library of Theses & Dissertations: http://www.ndltd.org/
- EThOS – British Library Electronic Theses Online: http://ethos.bl.uk/Home.do
- DEEP – DART Europe: http://www.dart-europe.eu

Grey Literature
- PAIS International: http://search.proquest.com/pais?accountid=8018 (via ProQuest)
- Open Grey: http://www.opengrey.eu/
- Mednar: http://mednar.com/mednar/desktop/en/green/search.html, one-stop federated search engine designed for professional medical researchers
- WorldwideScience: http://worldwidescience.org/

For peer review only - http://bmjopen.bmj.com/site/about/guidelines.xhtml

PRISMA-P (Preferred Reporting Items for Systematic review and Meta-Analysis Protocols) 2015 checklist: recommended items to address in a systematic review protocol*

Section and topic	Item No	Checklist item	Reported on Page
**ADMINISTRATIVE INFORMATION**			**1**
Title:			1
Identification	1a	Identify the report as a protocol of a systematic review	1
Update	1b	If the protocol is for an update of a previous systematic review, identify as such	
Registration	2	If registered, provide the name of the registry (such as PROSPERO) and registration number	1
Authors:			1
Contact	3a	Provide name, institutional affiliation, e-mail address of all protocol authors; provide physical mailing address of corresponding author	1
Contributions	3b	Describe contributions of protocol authors and identify the guarantor of the review	16
Amendments	4	If the protocol represents an amendment of a previously completed or published protocol, identify as such and list changes; otherwise, state plan for documenting important protocol amendments	
Support:			
Sources	5a	Indicate sources of financial or other support for the review	2
Sponsor	5b	Provide name for the review funder and/or sponsor	16
Role of sponsor or funder	5c	Describe roles of funder(s), sponsor(s), and/or institution(s), if any, in developing the protocol	16
**INTRODUCTION**			**4-7**
Rationale	6	Describe the rationale for the review in the context of what is already known	4-5
Objectives	7	Provide an explicit statement of the question(s) the review will address with reference to participants, interventions, comparators, and outcomes (PICO)	6-7
**METHODS**			**7-14**
Eligibility criteria	8	Specify the study characteristics (such as PICO, study design, setting, time frame) and report characteristics (such as years considered, language, publication status) to be used as criteria for eligibility for the review	10-11
Information sources	9	Describe all intended information sources (such as electronic databases, contact with study authors, trial registers or other grey literature sources) with planned dates of coverage	9
Search strategy	10	Present draft of search strategy to be used for at least one electronic database, including planned limits, such that it could be repeated	9-10
Study records:			7-14
Data management	11a	Describe the mechanism(s) that will be used to manage records and data throughout the review	7-8
Selection process	11b	State the process that will be used for selecting studies (such as two independent reviewers) through each phase of the review (that is, screening, eligibility and inclusion in meta-analysis)	11-14
Data collection process	11c	Describe planned method of extracting data from reports (such as piloting forms, done independently, in duplicate), any processes for obtaining and confirming data from investigators	13-14
Data items	12	List and define all variables for which data will be sought (such as PICO items, funding sources), any pre-planned data assumptions and	10-11

For peer review only

		simplifications	
Outcomes and prioritization	13	List and define all outcomes for which data will be sought, including prioritization of main and additional outcomes, with rationale	14
Risk of bias in individual studies	14	Describe anticipated methods for assessing risk of bias of individual studies, including whether this will be done at the outcome or study level, or both; state how this information will be used in data synthesis	14-15
Data synthesis	15a	Describe criteria under which study data will be quantitatively synthesised	14-15
	15b	If data are appropriate for quantitative synthesis, describe planned summary measures, methods of handling data and methods of combining data from studies, including any planned exploration of consistency (such as I^2, Kendall's τ)	N/A
	15c	Describe any proposed additional analyses (such as sensitivity or subgroup analyses, meta-regression)	N/A
	15d	If quantitative synthesis is not appropriate, describe the type of summary planned	15
Meta-bias(es)	16	Specify any planned assessment of meta-bias(es) (such as publication bias across studies, selective reporting within studies)	14-15
Confidence in cumulative evidence	17	Describe how the strength of the body of evidence will be assessed (such as GRADE)	14-15

*** The copyright for PRISMA-P (including checklist) is held by the PRISMA-P Group and is distributed under a Creative Commons Attribution Licence 4.0.**

From: Shamseer L, Moher D, Clarke M, Ghersi D, Liberati A, Petticrew M, Shekelle P, Stewart L, PRISMA-P Group. Preferred reporting items for systematic review and meta-analysis protocols (PRISMA-P) 2015: elaboration and explanation. BMJ. 2015 Jan 2;349(jan02 1):g7647.

For peer review only

Appendix 1: Enhancing transparency in reporting the synthesis of qualitative research: the ENTREQ statement

No.	Item	Guide and description	Reported on Page
1	Aim	State the research question the synthesis addresses.	6-7
2	Synthesis methodology	Identify the synthesis methodology or theoretical framework which underpins the synthesis, and describe the rationale for choice of methodology (e.g. meta-ethnography, thematic synthesis, critical interpretive synthesis, grounded theory synthesis, realist synthesis, meta-aggregation, meta-study, framework synthesis).	7-14
3	Approach to searching	Indicate whether the search was pre-planned (comprehensive search strategies to seek all available studies) or iterative (to seek all available concepts until they theoretical saturation is achieved).	9
4	Inclusion criteria	Specify the inclusion/exclusion criteria (e.g. in terms of population, language, year limits, type of publication, study type).	10-11
5	Data sources	Describe the information sources used (e.g. electronic databases (MEDLINE, EMBASE, CINAHL, psycINFO, Econlit), grey literature databases (digital thesis, policy reports), relevant organisational websites, experts, information specialists, generic web searches (Google Scholar) hand searching, reference lists) and when the searches conducted; provide the rationale for using the data sources.	9-10
6	Electronic Search strategy	Describe the literature search (e.g. provide electronic search strategies with population terms, clinical or health topic terms, experiential or social phenomena related terms, filters for qualitative research, and search limits).	9-10 Appendix 1
7	Study screening methods	Describe the process of study screening and sifting (e.g. title, abstract and full text review, number of independent reviewers who screened studies).	10-11
8	Study characteristics	Present the characteristics of the included studies (e.g. year of publication, country, population, number of participants, data collection, methodology, analysis, research questions).	N/A
9	Study selection results	Identify the number of studies screened and provide reasons for study exclusion (e,g, for comprehensive searching, provide numbers of studies screened and reasons for exclusion indicated in a figure/flowchart; for iterative searching describe reasons for study exclusion and inclusion based on modifications t the research question and/or contribution to theory development).	11-13
10	Rationale for appraisal	Describe the rationale and approach used to appraise the included studies or selected findings (e.g. assessment of conduct (validity and robustness), assessment of reporting (transparency), assessment of content and utility of the findings).	11-12

For peer review only

11	Appraisal items	State the tools, frameworks and criteria used to appraise the studies or selected findings (e.g. Existing tools: CASP, QARI, COREQ, Mays and Pope [25]; reviewer developed tools; describe the domains assessed: research team, study design, data analysis and interpretations, reporting).	11-13
12	Appraisal process	Indicate whether the appraisal was conducted independently by more than one reviewer and if consensus was required.	13
13	Appraisal results	Present results of the quality assessment and indicate which articles, if any, were weighted/excluded based on the assessment and give the rationale.	N/A
14	Data extraction	Indicate which sections of the primary studies were analysed and how were the data extracted from the primary studies? (e.g. all text under the headings "results /conclusions" were extracted electronically and entered into a computer software).	14
15	Software	State the computer software used, if any.	14
16	Number of reviewers	Identify who was involved in coding and analysis.	11-13
17	Coding	Describe the process for coding of data (e.g. line by line coding to search for concepts).	14
18	Study comparison	Describe how were comparisons made within and across studies (e.g. subsequent studies were coded into pre-existing concepts, and new concepts were created when deemed necessary).	14
19	Derivation of themes	Explain whether the process of deriving the themes or constructs was inductive or deductive.	14
20	Quotations	Provide quotations from the primary studies to illustrate themes/constructs, and identify whether the quotations were participant quotations of the author's interpretation.	14
21	Synthesis output	Present rich, compelling and useful results that go beyond a summary of the primary studies (e.g. new interpretation, models of evidence, conceptual models, analytical framework, development of a new theory or construct).	14-15

Extracted from: Tong A, Flemming K, McInnes E, et al. Enhancing transparency in reporting the synthesis of qualitative research: ENTREQ. ***BMC Medical Research Methodology*** **2012;12(1):181. doi: 10.1186/1471-2288-12-181 1**

For peer review only

BMJ Open

What are the experiences of seeking, receiving and providing FGM-related healthcare? Perspectives of health professionals and women / girls who have undergone FGM; Protocol for a systematic review of qualitative evidence

Journal:	*BMJ Open*
Manuscript ID	bmjopen-2017-018170.R1
Article Type:	Protocol
Date Submitted by the Author:	25-Aug-2017
Complete List of Authors:	Evans, Catrin; University of Nottingham School of Health Sciences, School of Health Sciences<br>Tweheyo, Ritah; University of Nottingham School of Health Sciences, School of Health Sciences<br>McGarry, Julie; University of Nottingham,<br>Eldridge, Jeanette; University of Nottingham School of Health Sciences, Research and Learning Services,<br>McCormick, Carol; Queen's Medical Centre<br>Nkoyo, Valentine; Mojatu Foundation<br>Higginbottom, Gina; University of Nottingham School of Health Sciences
**Primary Subject Heading**:	Evidence based practice
Secondary Subject Heading:	Public health, Evidence based practice, Qualitative research, Health services research, Health policy
Keywords:	Female Genital Mutilation/Cutting, qualitative evidence synthesis, immigrant health, health professional perspectives, migrant women and girls, systematic review

SCHOLARONE™ Manuscripts

What are the experiences of seeking, receiving and providing FGM-related healthcare? Perspectives of health professionals and women / girls who have undergone FGM; Protocol for a systematic review of qualitative evidence

Authors:
Dr. Catrin Evans (**corresponding author**)
Associate Professor, and Director of Global Engagement
School of Health Sciences, University of Nottingham, Queens Medical Centre, Nottingham, NG7 2HA. Email: Catrin.Evans@nottingham.ac.uk
orcid.org/0000-0002-5338-2191

Dr. Ritah Tweheyo
Research Fellow, School of Health Sciences, University of Nottingham, Queens Medical Centre, Nottingham, NG7 2HA. Email: Ritah.Tweheyo@nottingham.ac.uk
orcid.org/0000-0002-4609-7253

Dr. Julie McGarry
Associate Professor and Chair of the Domestic Violence and Abuse Integrated Research Group, School of Health Sciences, University of Nottingham, NG7 2HA
orcid.org/0000-0002-7629-2447

Jeanette Eldridge
Senior Research Librarian- Medicine & Health Sciences, Library Research & Learning Services, University of Nottingham, NG7 2RD
orcid.org/0000-0001-7341-4587

Carol McCormick
FGM Specialist Midwife, Nottingham University Hospital Trust

Valentine Nkoyo
Director- Mojatu Foundation, Founder of Nottingham Community FGM Steering Group and FGM Survivors Club

Prof. Gina Marie Awoko Higginbottom MBE,
The Mary Seacole Professor of Ethnicity and Community Health
School of Health Sciences, University of Nottingham Queens Medical Centre, Nottingham, NG7 2HA
orcid.org/0000-0001-8851-0985

Word Count: 3,393 words

For peer review only

Abstract

Introduction

Female Genital Mutilation (FGM) is an issue of global concern. High levels of migration mean that healthcare systems in higher-income western countries are increasingly being challenged to respond to the care needs of affected communities. Research has identified significant challenges in the provision of, and access to, FGM-related healthcare. There is a lack of confidence and competence among health professionals in providing appropriate care, suggesting an urgent need for evidence-based service development in this area. This study will involve two systematic reviews of qualitative evidence to explore the experiences, needs, barriers and facilitators to seeking and providing FGM-related healthcare in high income (OECD) countries, from the perspectives of: (1) women and girls who have undergone FGM and (2) health professionals.

Review methods

Twelve databases including MEDLINE, EMBASE, PsycINFO, ASSIA, Web of Science, ERIC, CINAHL, and POPLINE will be searched. Relevant grey literature will be identified from digital sources and professional networks.

Two reviewers will independently screen, select and critically appraise the studies. Study quality will be assessed using the JBI-QARI appraisal tool. Findings will be extracted into NVivo software. Synthesis by inductive thematic analysis, including in-depth reading, line by line coding of the findings, development of descriptive themes and re-coding to higher level analytical themes. Confidence in the review findings will be assessed using the GRADE-CERQual approach. Findings will be integrated into a comprehensive set of recommendations for research, policy and practice.

Dissemination

The syntheses will be reported as per the ENTREQ statement. Two reviews will be published in peer-reviewed journals and an integrated report disseminated at stakeholder engagement events.

Registration

The study is registered with the international prospective register of systematic reviews (PROSPERO): CRD42015030001:2015 and CRD42015030004: 2015.

Funder

The National Institute Health Research (NIHR) Health Service Research and Delivery Programme (NIHR 1115/137/04).

The researchers have no conflicts of interest.

For peer review only

Strengths and Limitations of this Study:

- Will illuminate health professional and organisational factors that influence choices and behaviour on providing FGM-care and following FGM management protocols in a range of healthcare settings (not just maternity settings).
- Will develop an in-depth understanding of women's/girl's care seeking choices, barriers and experiences across the life course (not just maternity settings).
- Will synthesize research in similar high-income contexts so that findings can be directly translated into interventions and service initiatives across OECD countries that share similar challenges in service provision and uptake.
- This is a participatory collaborative project that involves community representatives from identification of the initial questions through to dissemination.
- The systematic reviews will only include qualitative empirical evidence. Hence, a limitation is that opinion pieces, editorials and case studies of personal or professional experiences will be excluded.

Key words:

FGM, Female Genital Mutilation/Cutting, systematic review, qualitative evidence synthesis, migrant women and girls, FGM-related healthcare, health professional perspectives, immigrant health, OECD countries.

For peer review only

For peer review only - http://bmjopen.bmj.com/site/about/guidelines.xhtml

Introduction

Female Genital Mutilation (FGM) refers to all procedures that involve the partial or total removal of the external female genitalia or other injury to the female genital organs for non-medical reasons [1]. The practice is illegal in most countries and is an internationally recognised violation of the rights of women and girls [2]. FGM is associated with significant negative physical, psychological and sexual health sequelae [3]. In the immediate and short term, these include shock, infection, urinary retention or injury to other tissues (e.g. vaginal fistulae) [4 5]. In the longer term, they can include psychological problems, post-traumatic stress disorder, painful intercourse and other sexual problems, relationship problems, chronic pain, chronic infections, infertility and complications in childbirth [1 5]. It is essential therefore that affected women and girls have access to high-quality services that can identify and meet these multiple complex health needs, and that include mental as well as physical healthcare provision [1 6 7].

There are 30 countries where FGM is traditionally practised, with over 200 million women and girls affected worldwide, mainly from Africa and parts of Asia [2]. However, increased migration means that FGM is now regularly seen within health services in higher income ‘receiving’ countries where refugees and migrants from practising countries have settled [2 8]. It has been estimated that over half a million women and girls residing in European Union countries have FGM [8]. Within the UK, it is thought that approximately 137,000 women and girls living in England and Wales have undergone FGM [9], and that, since 2008, women with FGM make up approximately 1.5% (nearly 11,000) of all maternity episodes [9]. Hence, health services and health professionals in receiving countries are increasingly being challenged to develop appropriate services and to care for women/girls who have undergone FGM in a culturally sensitive manner [6 10]. Migrant’s access to healthcare in these countries can be problematic however, due to a range of socio-economic, legal, language and cultural barriers [11 12]. The ways in which these factors intersect to affect appropriate care delivery for women/girls who have undergone FGM – a particularly sensitive and personal issue - is currently unclear.

Existing evidence on women’s and health professionals’ experiences around FGM is primarily orientated to maternity care delivery, with much less attention devoted to care delivery in other settings, or for other problems or at other stages of the life course [1 13]. The available evidence from women however highlights a number of concerns, including not knowing about specialist services, feeling unable to talk about FGM, feeling judged and

For peer review only - http://bmjopen.bmj.com/site/about/guidelines.xhtml

experiencing fear, pain and isolation [14-22]. Likewise, evidence from health professionals indicates that they lack confidence and competence in caring for, and talking about, FGM [10 23-26]. Interestingly, even where training has been given and where clear care pathways and protocols exist, problems may still remain. For example, a recent study of FGM management in a large London maternity unit found that, in spite of the existence of guidelines and training, clinical care for women/girls with FGM was sub-optimal [27]. The maternity unit had access to a FGM specialist service, but 41% of women with FGM were not identified until they arrived in the labour ward. Hence, even though a specialist service existed, it was not being optimally used to benefit women with FGM, and a significant percentage of opportunities were missed to provide women with specialist care. Similar findings were reported from a study in a maternity unit in Switzerland where, in spite of staff training and the existence of clear guidelines, FGM was correctly identified and managed in only 34 (26.4%) of 129 cases reviewed [28]. The reasons for this lack of adherence to protocols are unclear however and there is a need to explore in more depth how organizational and personal factors may influence health professionals' views and behaviour in this area [10 24 25 29-36].

In the UK, a range of policies and protocols around FGM prevention and care have been developed over the last decade. These set out safeguarding procedures to mitigate risk of FGM and recommend a multi-agency approach to service development [6 37]. In the UK, these include; a declaration to end FGM in 2014, the establishment of a National FGM Prevention Programme, development of inter-collegiate guidelines [38-40], mandatory recording using the SCCI2026 FGM Enhanced Dataset Information Standard, the Serious Crime Act 2015, and mandatory reporting of girls at risk of FGM [41]. Many other OECD countries have similar laws barring FGM, including direct prosecution of suspects continuing the practice [37 42]. All EU countries consider FGM a crime of serious bodily harm, with legal measures to safeguard minors, and professional provisions for disclosure and duty to report [37]. Approaches to prosecution are varied, resulting in calls for efforts to improve and correct the inadequate care of women and girls who have undergone FGM [43], specifically for multidisciplinary approaches [13 44]. It is currently unknown how these legal and regulatory provisions impact upon on the experience of seeking and providing healthcare from patients' and health professionals' perspectives.

Much of the existing body of research around FGM relates to understanding the practice of FGM [45], the prevention of FGM [46 47] and the psycho-social and clinical consequences of

For peer review only - http://bmjopen.bmj.com/site/about/guidelines.xhtml

FGM [3 5 48 49]. Evidence on the effectiveness of FGM specific interventions or models of care is currently lacking [50 51], as concluded by a series of 10 systematic reviews published by WHO in a recent journal supplement [52]. In the absence of high quality evidence on the clinical or cost-effectiveness of different types of service provision, decision making around FGM services must be informed by other forms of evidence [53]. Hence, this project proposes to bring together existing qualitative evidence on lay and professional experiences of, and perspectives on, FGM care, and to relate this to service development in higher income (receiving) countries. In doing so, it will provide a vital resource for evidence-informed FGM-related service development and training. Qualitative research can provide insights into factors influencing service acceptability, appropriateness and meaningfulness, and on micro and macro level organizational and contextual issues that influence service use and delivery [54-56]. Therefore, the proposed qualitative evidence syntheses around FGM will generate rich data and deep understanding with which to inform new service initiatives and the content and structure of staff training resources [57 58]. The above mentioned WHO systematic review series [50] included 3 qualitative reviews, but these were focused on evidence related only to specific interventions, rather than healthcare experiences more generally, and hence identified a very limited number of studies [59-61].

There are a number of systematic reviews related to FGM healthcare that have been published to date, but all have taken a multi-context (or 'lumping') approach to the evidence [13 50 62-64], by including research from high and low-income settings across the world. Many key themes from these reviews therefore are drawn from evidence from very different contexts (see [52]) and are not easily transferable to a high-income setting. These countries generally have strong, well-resourced public health systems and FGM is primarily found within their migrant populations [2]. FGM care and its challenges in these settings, will therefore, be linked to other challenges around providing care for migrant populations (such as lack of familiarity with cultural norms, systems or communication issues [11]). This is a very different situation compared with countries where health systems are weak, where FGM is more prevalent, where health providers may have greater exposure to, and indeed, may even be complicit in, FGM (i.e. medicalisation of FGM [65]). In order to address this shortcoming, the proposed systematic reviews of women's and health professionals' experiences will focus exclusively on evidence from high income OECD contexts.

For peer review only - http://bmjopen.bmj.com/site/about/guidelines.xhtml

Aim

The aim of this review will be two-fold: to understand the experiences, needs, barriers and facilitators around seeking and providing FGM-related care in OECD countries from the perspectives of i) women and girls who have undergone FGM, and ii) health professionals. Hence, this will involve 2 reviews of qualitative evidence:

Review 1: ***To explore the experiences of FGM-related healthcare in OECD countries across the life course for women and girls who have undergone FGM.***

Objectives:

From the perspective of women and girls who have undergone FGM: -

i. to illuminate factors that influence FGM-related healthcare seeking and access to health services across the lifecourse,
ii. to explore how quality of care is perceived and experienced in different healthcare settings and with different groups of healthcare professionals,
iii. to characterise and explain elements of service provision considered important for the provision of acceptable and appropriate healthcare,
iv. to describe factors perceived to influence open discussion and communication around FGM (including prevention) with health professionals.

Review 2: ***To explore the views on, and experiences of, all cadres of health professionals in providing care across the lifecourse in OECD countries for women/girls who have undergone FGM.***

Objectives:

From the perspective of health professionals:

i. to explore how quality of care for women/girls who have undergone FGM is perceived in different healthcare settings and amongst different professional groups,
ii. to characterise and explain elements of service provision considered important for the provision of high quality care to women/girls who have undergone FGM,
iii. to illuminate factors perceived to facilitate or hinder appropriate provision of care for women and girls who have undergone FGM,

For peer review only - http://bmjopen.bmj.com/site/about/guidelines.xhtml

iv. to identify processes and practices perceived to influence open discussion and communication around FGM (including prevention) with women/girls from affected communities.

Methodology and Methods

The review methodology uses a participatory approach in which its aims and objectives were generated together with community organisations working in the field of FGM who continue to be involved as co-investigators and advisors [66]. The project originated from a request by a community organisation run by, and working with, FGM-affected women, to explore women's and health professionals' experiences in the healthcare encounter related to FGM. The review team and advisory group includes members of FGM-affected communities and their perspectives will be integrated into project activities at every stage, for example, in identifying relevant grey literature, contributing to interpretation of key themes, helping to formulate recommendations and helping to access professional and community networks to aid dissemination and community engagement.

The reviews seek to identify insights about lay/health professional experiences of FGM-related healthcare and perceived appropriateness and acceptability of services. These are questions best answered by qualitative research [54 55] – hence the specific focus on qualitative evidence. There are many possible approaches to qualitative evidence synthesis, with most discussions in this area characterizing the different types along a continuum between aggregation and interpretation [67]. Where the purpose of a synthesis is to generate new theoretical insights, a highly interpretive approach such as meta-ethnography may be most suitable, informed by an idealist epistemological stance [68 69]. However, where the purpose is to inform policy or practice a more aggregative or thematic approach informed by a realist epistemology is often advocated [69]. The latter is also suggested in cases where the existing evidence is likely to be descriptive (as in much health services research) rather than highly theoretical or conceptual [57]. An initial scoping of the literature suggests that this is the case for the proposed syntheses. A thematic synthesis approach involves using thematic analysis techniques to identify key concepts/themes within primary research studies [70 71]. Synthesis involves an iterative and inductive process of grouping themes into overarching categories and exploring the similarities, differences and relationships between them [57 58]. Thematic synthesis explicitly aims to move beyond generating a list of descriptive themes (as would be the case in meta-aggregation) in order to identify new, higher order, analytical insights that can contribute to new understandings of a phenomenon [57]. Review

For peer review only - http://bmjopen.bmj.com/site/about/guidelines.xhtml

recommendations however are clearly formulated to inform policy and practice and to identify research gaps. As such, it is considered the most suitable approach for the two proposed systematic reviews [72].

The findings of the two reviews will be reported separately and then, where appropriate, integrated (Fig.1) to enable greater understanding of key issues or concepts when presented from multiple perspectives. The findings will be brought together into a comprehensive set of recommendations for service development, community engagement, health professional education and future research.

Approach to searching

The project will implement a comprehensive search strategy to gather all available and accessible studies, including peer reviewed articles and grey literature. Primary research articles in the form of journal papers, research/evaluation reports, theses and dissertations will be collected. Reference lists from primary papers and key reviews will be hand searched to identify additional papers. There will be no language or date limits.

Data Sources

Searching will be done in at least 12 databases to include Medline (Ovid), Embase, Scopus, PsycINFO, Web of Science, ASSIA, CINAHL, ERIC, MIDIRS using a detailed search strategy (Appendix 1). Additional searches will be carried out in relevant indexes such as POPLINE, grey literature databases including: British library, NICE evidence services, index to theses, Networked Digital Library of Theses and Dissertations (NDLTD), ProQuest and other accessible digital thesis and dissertation repositories.

Search Strategy

The search strategy was developed from an initial scoping search undertaken to establish relevant search terms and potential databases and to develop a robust integrated, but specific and sensitive strategy. It is vital that we have a comprehensive search strategy because of known poor indexing of qualitative studies. Therefore, the scoping search will identify records which will be cross checked for indexing, together with FGM reports and guidelines from WHO to ensure correct terms and FGM-related terms are captured. The Librarian (JE) together with RT will develop and conduct all the searches. The comprehensive search will be tailored to each of the listed databases for both peer reviewed

For peer review only

For peer review only - http://bmjopen.bmj.com/site/about/guidelines.xhtml

and grey literature and results reported in a flowchart following PRISMA guidance. The scoping search will run from April to June 2017, and the expanded search will run from July to October 2017.

Electronic searches are summarised in Fig 2 and an initial detailed search strategy is attached in Appendix 1.

Screening and Eligibility

All returned results from the searches will be entered into the EndNote reference manager programme. Screening will be undertaken independently by 2 reviewers using title and abstract, and studies selected if they meet the a-priori selection criteria (see table 1). In case of disagreement, there will be recourse to a 3rd reviewer when required.

The inclusion criteria are:

- **Population:** Women and girls who have undergone FGM, and their experience of FGM-related healthcare, or healthcare professionals or students involved in the care of women and girls that have undergone FGM.
- **Phenomenon of Interest:** Experiences of seeking, receiving or providing FGM-related healthcare across the life course.
- **Context or Setting:** Studies conducted in OECD/high-income countries as described by the World Bank (List attached in Appendix 1).
- **Study type:** Any type of qualitative study and any type of mixed methods study that reports qualitative findings.
- **Search Limits:** No language or date limits. The abstracts of articles not in English will be translated and assessed. Those that meet the screening criteria will then be professionally translated into English for appraisal and data extraction.
- **Exclusion:** Any papers or research that was not undertaken on women and girls who have undergone FGM, or healthcare professionals, or not in OECD countries. Studies will be excluded if they do not describe FGM-related experiences of healthcare or provision of healthcare.

Table 1: *Screening Checklist*

Screening Criteria	Yes	No	Unsure
Does the study report findings related to……………… • Experiences of seeking and receiving **healthcare** related to FGM? • Experiences of providing **healthcare** to women/girls who			

For peer review only - http://bmjopen.bmj.com/site/about/guidelines.xhtml

have undergone FGM?			
Is the study conducted in an OECD country?			
Is it a qualitative study?<br>Or<br>Does it report qualitative findings from a mixed methods study?			
Are the participants…………………<br>• Women or girls who have undergone FGM?<br>• Health professionals?			

Quality Appraisal of Studies:

The role of critical appraisal in qualitative evidence synthesis is contested and there is lack of agreement over: (i) the appropriateness of excluding studies, (ii) the potential impact (or not) of excluding eligible papers on review outcomes, and, (iii) the criteria on which quality should be established [68 73-75]. For these reasons, the project will adopt an inclusive approach to critical appraisal, using the appraisal process to enable an in-depth understanding of each paper and to facilitate a critical, questioning approach to the study findings [76]. Studies will not be excluded on the basis of quality, rather, the quality assessment will be used: (i) to judge the relative contribution of each study to the overall synthesis, and, (ii) to assess the methodological rigour of each study as part of a process of assessing confidence in the review findings [77]. The quality of included studies will be assessed independently by 2 reviewers using the Joanna Briggs Institute Qualitative Assessment and Review Instrument (JBI-QARI) [78 79]. This tool has been found to be more coherent and more sensitive to assessment of validity than other commonly used tools [75]. An extract summary of the appraisal items is listed in Table 2.

Data extraction

Data extraction will primarily be undertaken by one reviewer with a 2nd and 3rd reviewer each checking a random sample of the articles for completeness in extraction. Data will be extracted using a modified JBI data extraction form (Table 3), to include details of the phenomenon of interest, population, context, study methodology, methods, and findings of significance to the review objectives [78 80]. If studies lack relevant detail, authors will be contacted to provide further information. The 'results/findings' sections from each study will be entered into NVivo® for further analysis and synthesis. Where a paper presents findings on both women's *and* health professional's experiences, the relevant sections will be extracted for each review separately.

For peer review only - http://bmjopen.bmj.com/site/about/guidelines.xhtml

***Table 2**: Extracted JBI Quality Appraisal Tool*

Summary JBI Qualitative Assessment and Review Instrument (QARI)													
**Record details / Full reference**	Is there congruity between the stated philosophical perspective and the research methodology?	Is there congruity between the research methodology and the research question or objectives?	Is there congruity between the research methodology and the methods used to collect data?	Is there congruity between the research methodology and the representation and analysis of data?	Is there congruity between the research methodology and the interpretation of results?	Is there a statement locating the researcher culturally or theoretically?	Is the influence of the researcher on the research, and vice-versa, addressed?	Are participants, and their voices, adequately represented?	Is the research ethical according to current criteria or, is there evidence of ethical approval by an appropriate body?	Do the conclusions drawn in the research report flow from the analysis, or interpretation, of the data?	**Score**	Seek further info	Comments (Including reason for exclusion)
	**Q1**	**Q2**	**Q3**	**Q4**	**Q5**	**Q6**	**Q7**	**Q8**	**Q9**	**Q10**	/10		

For peer review only - http://bmjopen.bmj.com/site/about/guidelines.xhtml

Table 3: *Data Extraction tool to include all the results and findings sections of each included study*

Modified JBI data extraction form for FGM Systematic Review											
Study (Name and Authors)	Purpose / Phenomena of interest	Methodology (type, theory / framework)	Methods (type, recruitment and sampling)	Setting	Geographical (Country and Region)	Cultural	Participants (Age, relevant number, sample)	Data analysis	Findings/ Results	Authors Conclusion	Reviewers Comments

For peer review only - http://bmjopen.bmj.com/site/about/guidelines.xhtml

Synthesis

Analysis will be conducted by 2 reviewers in consultation with the research team. We shall carry out an inductive-thematic analysis for each review [57], conducted in 4 phases: (i) in-depth reading and immersion in the data, (ii) coding of findings that are connected to FGM-related healthcare, (iii) re-coding to higher level themes and (iv) interpretive synthesis. If appropriate, the latter stage may involve the development of models or frameworks to identify and display relationships between, and patterns within, the analytical themes. Where possible, the analytical themes will be formulated as 'directive' findings indicating clear messages and/or suggesting clear lines of action for policy and practice [78].

Assessment of Confidence

The review findings will be assessed using the GRADE-CERQUAL guidelines [77]. The assessment of confidence in the evidence for an individual review finding considers four elements: (i) methodological limitations (the extent to which there are problems in the design or conduct of primary studies that contributed to evidence of a review finding); (ii) relevance (the extent to which the body of evidence from the primary studies supporting a review finding is applicable to the context specified in the review question); (iii) coherence (whether the finding is well grounded in data from the primary studies and can provide a convincing explanation for pattern found in the data); and (iv) adequacy of data (an overall determination of the degree of richness and quantity of data supporting a review finding) [77:2-4]. Based on the assessment, each review finding will be assigned one of four levels of confidence: high, moderate, low and very low.

Presenting and reporting the review

Each review will be reported in accordance with the 'Enhancing Transparency in Reporting the Synthesis of Qualitative Research' (ENTREQ) statement [81], appropriate for qualitative evidence synthesis, which consists of 21 items- see Table 4.

Table 4: *Summary of the 21 items from the ENTREQ Statement*

No	Item
1	Aim
2	Synthesis methodology
3	Approach to searching
4	Inclusion criteria
5	Data sources

For peer review only - http://bmjopen.bmj.com/site/about/guidelines.xhtml

6	Electronic Search strategy
7	Study screening methods
8	Study characteristics
9	Study selection results
10	Rationale for appraisal
11	Appraisal items
12	Appraisal process
13	Appraisal results
14	Data extraction
15	Software
16	Number of reviewers
17	Coding
18	Study comparison
19	Derivation of themes
20	Quotations
21	Synthesis output

Dissemination

The 2 qualitative systematic reviews will be published separately in open-access, peer reviewed, international journals and an integrated report compiled and widely disseminated to FGM stakeholders through stakeholder engagement events, round-table discussions with different stakeholder groups, and tailored materials for each of the stakeholder groups (academic, health professionals, policy makers/commissioners, FGM communities and community organisations). We shall share the review findings report in the second quarter of 2018, using pre-existing contacts with the WHO Europe FGM group network and through professional networks.

The participatory collaborative approach to this qualitative evidence synthesis project will enhance the multidisciplinary interpretation of findings in order to inform stakeholders and the development of FGM-care initiatives for improved confidence and quality of service provision to women and girls who have undergone FGM.

Conflict of interest

The authors declare no competing interests in the development of this manuscript.

Acknowledgements

This systematic review is funded under Project: ***Improving Care for Women and Girls who have undergone Female Genital Mutilation (FGM): Qualitative Evidence Synthesis***.

For peer review only - http://bmjopen.bmj.com/site/about/guidelines.xhtml

Funded by the National Institute Health Research (NIHR) Health Service Research and Delivery Programme (NIHR 1115/137/04).

Author Contributions

CE conceptualised the project and prepared the project documents as PI in collaboration with the team members GH, JM, CC, and VN.

JE developed the search strategy and will carry out the searches

RT prepared the manuscript with input from team members.

All team members reviewed and approved this manuscript.

References

1. World Health Organisation. WHO guidelines on the management of health complications from Female Genital Mutilation. Geneva: WHO, 2016.
2. United Nations Children's Fund. Female Genital Mutilation/Cutting: A Global Concern. New York: UNICEF, 2016.
3. World Health Organisation. A Systematic Review of the Health Complications of Female Genital Mutilation Including Sequelae in Childbirth. Geneva: World Health Organisation, 2000.
4. Berg RC, Underland V, Odgaard-Jensen J, et al. Effects of female genital cutting on physical health outcomes: A systematic review and meta-analysis. *BMJ Open* 2014;4(11):no pagination.
5. Berg RC, Denison E, A. F. Psychological, social and sexual consequences of female genital mutilation/cutting (FGM/C): a systematic review of quantitative studies. Centre for Reviews and Dissemination. Oslo: Norwegian Knowledge Centre for the Health Services (NOKC), 2010.
6. HM Government. Multi-agency statutory guidance on Female Genital Mutilation. In: Department for Education, Department of Health, Home Office, eds. https://www.gov.uk/government/publications/multi-agency-statutory-guidance-on-female-genital-mutilation, 2016.
7. Creighton S, Hodes D. Female Genital Mutilation (FGM): Chapter 3: Annual Report of the Chief Medical Officer: The Health of the 51%: Women. In: Department of Health, ed. London: Department of Health, 2015.
8. Van Baelen L, Ortensi L, Leye E. Estimates of first-generation women and girls with female genital mutilation in the European Union, Norway and Switzerland. *The European Journal of Contraception & Reproductive Health Care* 2016;21(6):474-82.
9. Macfarlane A, Dorkenoo E. Prevalence of female genital mutilation in England and Wales: National and local estimates. London: City University, 2015.
10. Degni F, Suominen S, Essen B, et al. Communication and cultural issues in providing reproductive health care to immigrant women: health care providers' experiences in meeting the needs of [corrected] Somali women living in Finland. *Journal of Immigrant and Minority Health* 2012;14(2):330-43. doi: https://dx.doi.org/10.1007/s10903-011-9465-6
11. Sarría-Santamera A, Hijas-Gómez AI, Carmona R, et al. A systematic review of the use of health services by immigrants and native populations. *Public Health Reviews* 2016;37(1) doi: 10.1186/s40985-016-0042-3

For peer review only - http://bmjopen.bmj.com/site/about/guidelines.xhtml

12. Suphanchaimat R, Kantamaturapoj K, Putthasri W, et al. Challenges in the provision of healthcare services for migrants: a systematic review through providers' lens. *BMC Health Services Research* 2015;15:390. doi: 10.1186/s12913-015-1065-z
13. Zurynski Y, Sureshkumar P, Phu A, et al. Female Genital Mutilation and cutting: a systematic literature review of health professionals' knowledge, attitudes and clinical practice. *BMC International Health & Human Rights* 2015;15:32.
14. Berggren V, Bergstrom S, Edberg AK. Being different and vulnerable: Experiences of immigrant African women who have been circumcised and sought maternity care in Sweden. *Journal of Transcultural Nursing* 2006;17(1):50-57.
15. Chalmers B, Hashi KO. 432 Somali women's birth experiences in Canada after earlier female genital mutilation. *Birth* 2000;27(4):227-34.
16. Moxey JM, Jones LL. A qualitative study exploring how Somali women exposed to female genital mutilation experience and perceive antenatal and intrapartum care in England. *BMJ Open* 2016;6 (1) (no pagination)(e009846)
17. Vloeberghs E, Van der kwaak A, Knipscheer J, et al. Coping and chronic psychosocial consequences of female genital mutilation in the Netherlands. *Ethnicity & Health* 2012;17(6):677-95.
18. d'Entremont M, Smythe L, McAra-Couper J. The sounds of silence: A hermeneutic interpretation of childbirth post excision. *Health Care for Women International* 2014;35(3):300-19. doi: http://dx.doi.org/10.1080/07399332.2013.838245
19. Hussein E. Women's experiences, perceptions and attitudes of female genital mutilation: the Bristol PEER study: Foundation for Women's Health Research and Development (FORWARD), 2010.
20. Upvall MJ, Mohammed K, Dodge PD. Perspectives of Somali Bantu refugee women living with circumcision in the United States: a focus group approach. *International Journal of Nursing Studies* 2009;46(3):360-8. doi: 10.1016/j.ijnurstu.2008.04.009
21. Vangen S, Johansen REB, Sundby J, et al. Qualitative study of perinatal care experiences among Somali women and local health care professionals in Norway. *European Journal of Obstetrics & Gynecology & Reproductive Biology* 2004;112(1):29-35. doi: 10.1016/s0301-2115(03)00313-0
22. Recchia N, McGarry J. "Don't judge me": narratives of living with FGM. *Ethnicity and Inequalities in Health and Social Care* 2017;10(1):4-13.
23. Clayton-Hathway K. A pilot evaluation of health services for communities affected by FGM in Oxfordshire: Final report. Oxford: Oxford Against Cutting, 2016.
24. Kaplan-Marcusan A, Toran-Monserrat P, Moreno-Navarro J, et al. Perception of primary health professionals about female genital mutilation: from healthcare to intercultural competence. *BMC Health Services Research* 2009;9:11. doi: 10.1186/1472-6963-9-11
25. Dawson AJ, Turkmani S, Varol N, et al. Midwives' experiences of caring for women with female genital mutilation: Insights and ways forward for practice in Australia. *Women and Birth* 2015;28(3):207-14. doi: http://dx.doi.org/10.1016/j.wombi.2015.01.007
26. Johansen E. Care for infibulated women giving birth in Norway. *Medical Anthropology Quarterly* 2006;20(4):516-44.
27. Zenner N, Liao LM, Richens Y, et al. Quality of obstetric and midwifery care for pregnant women who have undergone female genital mutilation. *Journal of Obstetrics and Gynaecology* 2013;33(5):459-62. doi: 10.3109/01443615.2013.767785
28. Abdulcadir J, Dugerdil A, Boulvain M, et al. Missed opportunities for diagnosis of female genital mutilation. *International Journal of Gynaecology and Obstetrics* 2014;125(3):256-60. doi: 10.1016/j.ijgo.2013.11.016
29. Cappon S, L'Ecluse C, Clays E, et al. Female genital mutilation: Knowledge, attitude and practices of Flemish midwives. *Midwifery* 2015;31(3):e29-35 1p. doi: 10.1016/j.midw.2014.11.012

For peer review only - http://bmjopen.bmj.com/site/about/guidelines.xhtml

30. Fawcett L. Somali refugee women and their U.S. healthcare providers: Knowledge, perceptions and experiences of childbearing. *Dissertation Abstracts International Section A: Humanities and Social Sciences* 2015;75(8-A(E)):No Pagination Specified.
31. Isman E, Mahmoud Warsame A, Johansson A, et al. Midwives' Experiences in Providing Care and Counselling to Women with Female Genital Mutilation (FGM) Related Problems. *Obstetrics and Gynecology International* 2013;2013:785148. doi: 10.1155/2013/785148
32. Kaplan-Marcusan A, Del Rio NF, Moreno-Navarro J, et al. Female genital mutilation: perceptions of healthcare professionals and the perspective of the migrant families. *BMC Public Health* 2010;10:193. doi: 10.1186/1471-2458-10-193
33. León-Larios F, Casado-Mejía R. Knowledge, attitudes and professional experiences of midewives in primary health care in Seville on female genital mutilation. *Matronas Profesion* 2014;15(2):56-61 6p.
34. Tamaddon L, Johnsdotter S, Liljestrand J, et al. Swedish health care providers' experience and knowledge of female genital cutting. *Health Care Women International* 2006;27(8):709-22. doi: http://dx.doi.org/10.1080/07399330600817741
35. Widmark C, Leval A, Tishelman C, et al. Obstetric care at the intersection of science and culture: Swedish doctors' perspectives on obstetric care of women who have undergone female genital cutting. *Journal of Obstetrics and Gynaecology* 2010;30(6):553-58.
36. Widmark C, Tishelman C, Ahlberg BM. A study of Swedish midwives' encounters with infibulated African women in Sweden. *Midwifery* 2002;18(2):113-25.
37. Leye E, Deblonde J, García-Añón J, et al. An analysis of the implementation of laws with regard to female genital mutilation in Europe. *Crime, Law and Social Change* 2007;47(1):1-31. doi: http://dx.doi.org/10.1007/s10611-007-9055-7
38. Royal College of Midwives, Royal College of Nursing, Royal College of Obstetricians and Gynaecologists, et al. Intercollegiate guidelines tackling FGM in the UK: Intercollegiate recommendations for identifying, recording and reporting. London, 2013.
39. Royal College of Nursing. Female genital mutilation: An RCN resource for nursing and midwifery practice (2nd ed). London: RCN, 2015.
40. Royal College of Obstetricians and Gynaecologists. Green-top guideline no.53: Female genital mutilation and its management. London, 2015.
41. Department of Health. Female Genital Mutilation Risk and Safeguarding; Guidance for professionals. London: Crown copyright, 2016.
42. United States. Agency for International D. Female Genital Mutilation / cutting: United States Government's response: [Washington, D.C.], USAID, 2014 Jul 21., 2014:[3] p.
43. Rouzi AA, Alturki F. Female Genital Mutilation/cutting: an update. *Clinical and Experimental Obstetrics & Gynecology* 2015;42(3):300-3.
44. Creighton SM, Dear J, de Campos C, et al. Multidisciplinary approach to the management of children with female genital mutilation (FGM) or suspected FGM: service description and case series. *BMJ Open* 2016;6(2):e010311.
45. Berg RC, Denison E. A Tradition in Transition: Factors Perpetuating and Hindering the Continuance of Female Genital Mutilation/Cutting (FGM/C) Summarized in a Systematic Review. *Health Care for Women International* 2013;34(10):837-59.
46. Varol N, Turkmani S, Black K, et al. The role of men in abandonment of female genital mutilation: a systematic review. *BMC Public Health* 2015;15(1):1034. doi: 10.1186/s12889-015-2373-2
47. Berg RC, Denison EM. A realist synthesis of controlled studies to determine the effectiveness of interventions to prevent genital cutting of girls. *Paediatrics and International Child Health* 2013;33(4):322-33. doi: 10.1179/2046905513Y.0000000086
48. Mulango P, Hollins-Martin C, McAndrew S. The psychological impact of Female Genital Mutilation-Cutting (FGM-C) on girls-women's mental health a narrative literature review. *Journal of Reproductive and Infant Psychology* 2014;32(5) doi: 10.1080/02646838.2014.949641

For peer review only - http://bmjopen.bmj.com/site/about/guidelines.xhtml

49. Berg RC, Underland V. The obstetric consequences of female genital mutilation/cutting: a systematic review and meta-analysis. *Obstetrics & Gynecology International* 2013;2013:496564. doi: 10.1155/2013/496564
50. Sunday-Adeoye I, Serour G. Management of health outcomes of female genital mutilation: Systematic reviews and evidence syntheses. *International Journal of Gynaecology & Obstetrics* 2017;136 Suppl 1:1-2.
51. Berg RC, Taraldsen S, Said MA, et al. The effectiveness of surgical interventions for women with FGM/C: a Systematic Review. *BJOG: An International Journal of Obstetrics and Gynaecology* 2017 doi: 10.1111/1471-0528.14839
52. Stein K, Hindin JM. Management of health outcomes of female genital mutilation: Systematic reviews and evidence syntheses. *International Journal of Gynecology & Obstetrics* 2017;136,(Supplement 1,)
53. Abdulcadir J, Rodriguez MI, Say L. Research gaps in the care of women with female genital mutilation: An analysis. *British Journal of Obstetrics & Gynaecology* 2015;122(3):294-303. doi: 10.1111/1471-0528.13217
54. Popay J, Williams G. Qualitative research and evidence based healthcare. *Journal of the Royal Society of Medicine* 1998;35(91):32-37.
55. Pope C, Mays N. Reaching the parts other methods cannot reach: an introduction to qualitative methods in health and health services research. *British Medical Journal* 1995(6996):42-45.
56. Medical Research Council. Developing and evaluating complex interventions: new guidance. London 2008.
57. Thomas J, Harden A. Methods for the thematic synthesis of qualitative research in systematic reviews. *BMC Medical Research Methodology* 2009;8:45.
58. Ring N, Ritchie K, Mandava L, et al. A guide to synthesising qualitative research for researchers undertaking health technology assessment and systematic reviews. Edinburgh: NHS Quality Improvement Scotland and University of Stirling, 2011.
59. Smith H, Stein K. Health information interventions for female genital mutilation. *International Journal of Gynaecology & Obstetrics* 2017;136 Suppl 1:79-82.
60. Smith H, Stein K. Surgical or medical interventions for female genital mutilation. *International Journal of Gynaecology & Obstetrics* 2017;136 Suppl 1:43-46.
61. Smith H, Stein K. Psychological and counselling interventions for female genital mutilation. *International Journal of Gynaecology & Obstetrics* 2017;136 Suppl 1:60-64. doi: https://dx.doi.org/10.1002/ijgo.12051
62. Hannes K, Harden A. Multi-context versus context-specific qualitative evidence syntheses: combining the best of both. *Research Synthesis Methods* 2011;2(4):271-78. doi: 10.1002/jrsm.55
63. Weir MC, Grimshaw JM, Mayhew A, et al. Decisions about lumping vs. splitting of the scope of systematic reviews of complex interventions are not well justified: a case study in systematic reviews of health care professional reminders. *Journal of Clinical Epidemiology* 2012;65(7):756-63. doi: 10.1016/j.jclinepi.2011.12.012 [published Online First: 2012/04/14]
64. Berg RC, Taraldsen S, Said MA, et al. Reasons for and Experiences With Surgical Interventions for Female Genital Mutilation/Cutting (FGM/C): A Systematic Review. *Journal of Sexual Medicine* 2017;14(8):977-90. doi: 10.1016/j.jsxm.2017.05.016
65. Doucet M-H, Pallitto C, Groleau D. Understanding the motivations of health-care providers in performing female genital mutilation: an integrative review of the literature. *Reproductive Health* 2017;14(1):46.
66. Minkler M, Wallerstein N. Community Based Participatory Research. San Francisco: Jossey Bass 2003.
67. Barnett-Page E, Thomas J. Methods for the synthesis of qualitative research: a critical review. *BMC Medical Research Methodology* 2009;9:59.

For peer review only - http://bmjopen.bmj.com/site/about/guidelines.xhtml

68. Toye F, Seers K, Allcock N, et al. 'Trying to pin down jelly'-exploring intuitive processes in quality assessment for meta-ethnography. *BMC Medical Research Methodology* 2013;13(1):46.
69. Paterson B. "It looks great but how do I know it fits?": An introduction to meta-synthesis research. In: Hannes H, Lockwood C, eds. Synthesizing Qualitative Research: Choosing the Right Approach. Oxford: Wiley Blackwell BMJ Books 2012:1-21.
70. Boyatzis R. Transforming qualitative data: thematic analysis and code development. London: Sage 1998.
71. Braun V, Clarke V. Using thematic analysis in psychology. *Qualitative Research in Psychology* 2006;3(2):77-101.
72. Noyes J, Booth A, Hannes K, et al. Supplementary guidance for inclusion of qualitative research in Cochrane systematic reviews of interventions, Version 1: Cochrane Collaboration Qualitative Methods Group 2011.
73. Carroll C, Booth A, Lloyd-Jones M. Should We Exclude Inadequately Reported Studies From Qualitative Systematic Reviews? An Evaluation of Sensitivity Analyses in Two Case Study Reviews. *Qualitative Health Research* 2012;22(10):1425-34.
74. Cohen D, Crabtree B. Evaluative criteria for qualitative research in health care: controversies and recommendations. *Annals of Family Medicine* 2008;6:4.
75. Hannes K, Lockwood C, Pearson A. A comparative analysis of three online appraisal instruments' ability to assess validity in qualitative research. *Qualitative Health Research* 2010;20(12):1736-43. doi: 10.1177/1049732310378656 [published Online First: 2010/07/31]
76. Hannes K, Pearson A. Obstacles to the Implementation of Evidence Based Practice in Belgium: a Worked Example of Meta-Aggregation. In: Hannes K, Lockwood C, eds. Synthesizing Qualitative Research: Choosing the Right Approach: BMJ Books, Wiley Blackwell 2012:21-41.
77. Lewin S, Glenton C, Munthe-Kaas H, et al. Using qualitative evidence in decision making for health and social interventions: An approach to assess confidence in findings from qualitative evidence syntheses (GRADE-CERQUAL). *Plos Medicine* 2015;12(10):e1001895.
78. Pearson A, Robertson-Malt S, Rittenmeyer L. Synthesizing qualitative evidence. Philadelphia: Lippincott-Joanna Briggs Institute 2011.
79. The Joanna Briggs Institute. Critical Appraisal Checklist for Qualitative Research 2016 [Available from: http://joannabriggs.org/research/critical-appraisal-tools.html.
80. The Joanna Briggs Institute. JBI QARI Data Extraction Form for Interpretive and Critical Research 2016 [Available from: http://joannabriggs.org/assets/docs/jbc/operations/dataExtractionForms/JBC_Form_DataE_IntCrit.pdf.
81. Tong A, Flemming K, McInnes E, et al. Enhancing transparency in reporting the synthesis of qualitative research: ENTREQ. *BMC Medical Research Methodology* 2012;12(1):181. doi: 10.1186/1471-2288-12-181

For peer review only - http://bmjopen.bmj.com/site/about/guidelines.xhtml

Figure legends

Figure 1: Systematic reviews to improve FGM-related care

Figure 2: Search Summary flowchart

Tables:

Table 1: *Screening Checklist*

Table 2: *Extracted JBI Quality Appraisal Tool*

Table 3: *Data Extraction tool to include all the results and findings sections of each included study*

Table 4: *Summary of the 21 items from the ENTREQ Statement*

For peer review only

For peer review only - http://bmjopen.bmj.com/site/about/guidelines.xhtml

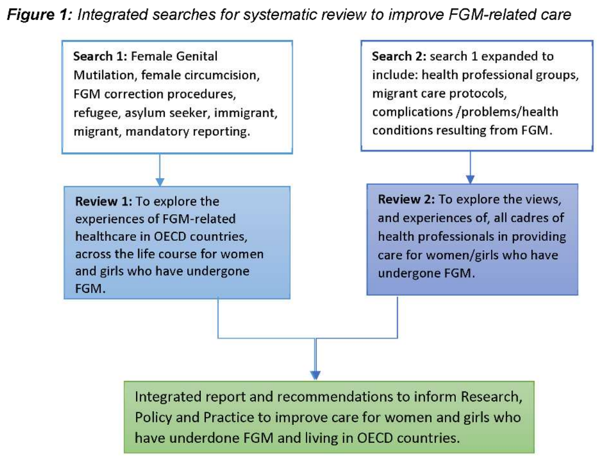

Figure 1: Systematic reviews to improve FGM-related care

209x297mm (300 x 300 DPI)

Figure 2: Search summary flowchart

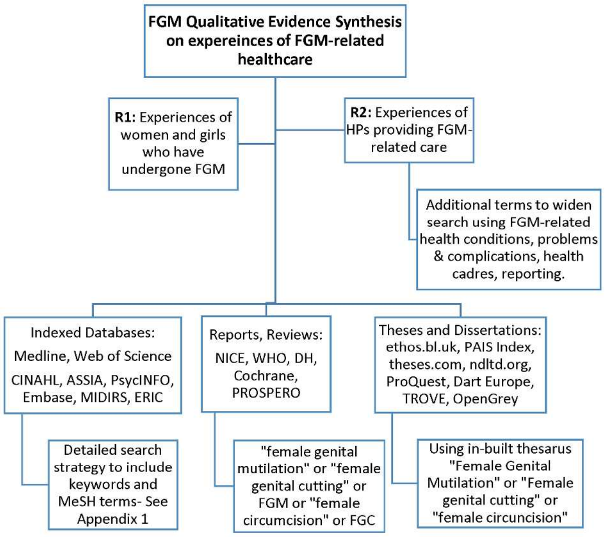

Figure 2: Search summary flowchart

209x297mm (300 x 300 DPI)

Appendix 1: Detailed Search Strategy

The search strategy was developed in order to meet the requirements for both systematic reviews:

- **R1-**To explore the experiences of FGM-related healthcare across the life course for women and girls who have undergone FGM.
- **R2-**To explore the views on, and experiences of, all cadres of health professionals in providing care for women/girls who have undergone FGM.

Search strategy in Ovid Medline

#	Search terms	Notes
1.	(exp Circumcision, Female/ or (exp Genitalia, Female/ and exp Medicine, Traditional/))	
2.	("female genital mutilation" or "female circumcision" or "female genital cutting" or "traditional female genital surgery" or infibulat* or reinfibulat* or defibulat* or clitoridect* or ((((ritual* or traditional* or ceremon* or sociali#ation*) adj3 (practic* or cut or cutting or surg*)) or circumcis* or excis*) adj3 (female* or wom#n or girl* or child* or adolescen*))).mp.	Different FGM-related terms
3.	((FGM or FGC or TFGS or FGMC or "FGM/C" or thara or tahar or khitan or khifad or khafad or megrez or absum or mekhnishab or kutairi or ibi or ugwu or sunna or bondo or sonde or gudiniin or halalays or qodiin or tahoor or bagne or gadja or ganza or "fanadu di mindjer" or niaka or kuyango or "musolula karoola").mp. or (egypt* or sudan* or ethiopi* or somali* or djibouti* or eritrea* or burkin* or guinea* or sierra leone or leonean* or mauritan* or mali or malian*).ti,ab.) adj3 (female* or wom#n or girl* or child* or adolescen*)	Expanded FGM terms (to include country specific name for the practice.
4.	1 or 2 or 3	
5.	"Emigrants and Immigrants"/ or Refugees/ or "Transients and Migrants"/ or "Emigration and Immigration"/ or exp Vulnerable Populations/ or exp Ethnic Groups/ or 31. exp african continental ancestry group/ or exp asian continental ancestry group/	Immigrant populations
6.	Limit 5 to female	
7.	(immigrant* or immigrat* or migrant* or migrat* or emigrant* or emigrat* or emigre* or expat* or ex-pat* or transients or newcomer* or new-comer* or alien* or incomer* or in-comer* or refugee* or (asylum adj seek*) or asylee* or (refused adj3 (asylum* or refugee*)) or (displaced adj person*) or exile* or “new arrival*” or (country adj2 (birth or origin)) or transnational* or foreigner* or (foreign adj (born or citizen* or national* or origin*)) or (non adj (citizen* or native*)) or ((adoptive or naturali#ed) adj (citizen* or resident*)) or overstay* or trafficked or "spousal migrant*" or ethnic* or ethno* or race or racial*).mp. or ((vulnerab* or disadvantag* or minorit*) adj3 (individ* or person* or people* or population* or communit* or group*)).ti,ab.	Expanded immigrant terms commonly used in OECD countries
8.	Limit 7 to female	
9.	("Black and Minority Ethnic" or "Black & Minority ethnic" or BME or BAME or black african*).mp. or<br>(africa* or "middle east*" or asia* or iran* or iraq* or israel* or oman* or "united arab emirat*" or UAE or "saudi arabia*" or palestin* or india* or indonesia* or malaysia* or pakistan* or egypt* or yemen* or sudan* or ethiopi* or somali* or djibouti* or eritrea* or kenya* or uganda* or	Expanded immigrant terms to include countries and populations

For peer review only - http://bmjopen.bmj.com/site/about/guidelines.xhtml

	tanzania* or camero?n* or chad* or niger* or benin* or togo* or ghana* or burkin* or ivory coast* or cote d'ivoire or liberia* or guinea* or sierra leone or leonean* or gambia* or senegal* or mauritan* or mali or malian* or "sara subgroup" or ngama).ti,ab.	where FGM is a common practice
10.	Limit 9 to female	
11.	6 or 8 or 10	
12.	exp Community Health Workers/ or exp Volunteers/ or exp Occupational Groups/ or exp Health Personnel/ or exp Midwifery/ or exp General Practitioners/ or exp Physicians/ or exp Nurses/ or exp Nurses, Community Health/ or exp Nurses' Aides/ or exp Nursing Staff, Hospital/ or exp Nursing Staff/	Health professional cadres
13.	(pregnan* or birth* or childbirth* or matern* or gyn#e* or obstetric* or menstru* or labo#r* or vulv* or vagina* or uter*).ti,ab.	Commonly reported in regard to FGM
14.	exp Pregnancy/ or exp Pregnancy Complications/ or exp Pregnancy High Risk/ or exp Obstetric Labor Complications/ or Female Urogenital Diseases/ or exp Menstruation Disturbances/ or exp Genital Diseases, Female/ or exp Vaginal Diseases/ or exp Vulvar Diseases/ or exp Vaginismus/ or exp Vulvodynia/ or exp Dyspareunia/ or exp Vaginitis/ or exp Vulvovaginitis/ or exp Vaginal Fistula/ or exp Rectovaginal Fistula/ or Genital Diseases, Female/ or exp Infertility, Female/	FGM-related conditions, problems, complications
15.	((pelvic* or back* or urolog* or urogenit* or urinat* or genit* or abdomin*) adj3 (poor* or adverse or complicat* or difficult* or disorder* or dysfunction* or disease* or pain* or risk* or danger* or problem* or issue* or concern* or infect* or inflamm*) adj3 (female* or wom#n or girl* or child* or adolescen*)).mp.	
16.	exp Pelvic Inflammatory Disease/ or exp Urologic Diseases/ or exp Pelvic Pain/ or exp Sexual Dysfunctions, Psychological/ or exp Pelvic Infection/ or exp Kidney Diseases/ or exp Urinary Fistula/ or exp Rectal Fistula/ or exp Urinary Bladder Fistula/	
17.	Limit 16 to female	
18.	((anxiet* or anxious* or depress* or self-harm* or self-injur* or suicid*) adj3 (female* or wom#n or girl* or child* or adolescen*)).mp.	Expanded to include mental health conditions
19.	exp Mental Disorders/ or exp Depressive Disorder, Major/ or exp Suicide/ or exp Depression/ or exp Suicidal Ideation/ or exp Depressive Disorder/ or exp Bipolar Disorder/ or exp Suicide, Attempted/ or exp Self-Injurious Behavior/ or exp Substance-Related Disorders/ or exp Social Isolation/	
20.	Limit 19 to female	
21.	((((sex* or viral* or virus* or bacteria*) adj3 diseas*) or HIV or STD* or AIDS) adj3 (female* or wom#n or girl* or child* or adolescen*)).mp.	
22.	exp Sexually Transmitted Diseases, Viral/ or exp Sexually Transmitted Diseases/	
23.	Limit 22 to female	
24	13 or 14 or 15 or 17 or 18 or 20 or 21 or 23	
25.	(AUSTRALIA* or AUSTRIA* or BELGIUM* or belgian* or CANAD* or CHILE* or CZECH* or DENMARK* or danish* or ESTONIA* or FINLAND or finnish or FRANCE or French or GERMAN* or GREECE or greek* or HUNGAR* or ICELAND* or IRELAND or irish or ISRAEL* or ITALY or italian* or JAPAN* or KOREA* or LATVIA* or LUXEMB* or MEXIC* or NETHERLAND* or holland* or dutch or "low countries" or europe* or "NEW ZEALAND*" or NORWAY or	OECD countries

For peer review only

	norwegian* or POLAND or polish or PORTUGAL or portuguese or SLOVAK* or SLOVENIA* or SPAIN or spanish or SWED* or SWITZERLAND or swiss or TURK* or "UNITED KINGDOM" or "UK" or britain or british or england or english or scot* or wales or welsh or UNITED STATES or "USA" or america*).mp.	
26.	randomized controlled trial.pt.	Study types not relevant for qualitative evidence
27.	controlled clinical trial.pt.	
28.	randomized.ab.	
29.	placebo.ab.	
30.	clinical trials as topic.sh.	
31.	randomly.ab.	
32.	trial.ti.	
33.	(animals not (humans and animals)).sh.	
34.	26 or 27 or 28 or 29 or 30 or 31 or 32	
35.	34 not 33	
36.	11 and 12 and 24	
37.	4 and 12	
38.	4 or 36 or 37	
39.	38 and 25	
40.	39 not 35	
41.	40 not 33	

For peer review only

PRISMA-P (Preferred Reporting Items for Systematic review and Meta-Analysis Protocols) 2015 checklist: recommended items to address in a systematic review protocol*

Section and topic	Item No	Checklist item	Reported on Page
**ADMINISTRATIVE INFORMATION**			**1**
Title:			1
Identification	1a	Identify the report as a protocol of a systematic review	1
Update	1b	If the protocol is for an update of a previous systematic review, identify as such	
Registration	2	If registered, provide the name of the registry (such as PROSPERO) and registration number	1
Authors:			1
Contact	3a	Provide name, institutional affiliation, e-mail address of all protocol authors; provide physical mailing address of corresponding author	1
Contributions	3b	Describe contributions of protocol authors and identify the guarantor of the review	16
Amendments	4	If the protocol represents an amendment of a previously completed or published protocol, identify as such and list changes; otherwise, state plan for documenting important protocol amendments	
Support:			
Sources	5a	Indicate sources of financial or other support for the review	2
Sponsor	5b	Provide name for the review funder and/or sponsor	16
Role of sponsor or funder	5c	Describe roles of funder(s), sponsor(s), and/or institution(s), if any, in developing the protocol	16
**INTRODUCTION**			**4-7**
Rationale	6	Describe the rationale for the review in the context of what is already known	4-5
Objectives	7	Provide an explicit statement of the question(s) the review will address with reference to participants, interventions, comparators, and outcomes (PICO)	6-7
**METHODS**			**7-14**
Eligibility criteria	8	Specify the study characteristics (such as PICO, study design, setting, time frame) and report characteristics (such as years considered, language, publication status) to be used as criteria for eligibility for the review	10-11
Information sources	9	Describe all intended information sources (such as electronic databases, contact with study authors, trial registers or other grey literature sources) with planned dates of coverage	9
Search strategy	10	Present draft of search strategy to be used for at least one electronic database, including planned limits, such that it could be repeated	9-10
Study records:			7-14
Data management	11a	Describe the mechanism(s) that will be used to manage records and data throughout the review	7-8
Selection process	11b	State the process that will be used for selecting studies (such as two independent reviewers) through each phase of the review (that is, screening, eligibility and inclusion in meta-analysis)	11-14
Data collection process	11c	Describe planned method of extracting data from reports (such as piloting forms, done independently, in duplicate), any processes for obtaining and confirming data from investigators	13-14
Data items	12	List and define all variables for which data will be sought (such as PICO items, funding sources), any pre-planned data assumptions and	10-11

For peer review only

		simplifications	
Outcomes and prioritization	13	List and define all outcomes for which data will be sought, including prioritization of main and additional outcomes, with rationale	14
Risk of bias in individual studies	14	Describe anticipated methods for assessing risk of bias of individual studies, including whether this will be done at the outcome or study level, or both; state how this information will be used in data synthesis	14-15
Data synthesis	15a	Describe criteria under which study data will be quantitatively synthesised	14-15
	15b	If data are appropriate for quantitative synthesis, describe planned summary measures, methods of handling data and methods of combining data from studies, including any planned exploration of consistency (such as I^2, Kendall's τ)	N/A
	15c	Describe any proposed additional analyses (such as sensitivity or subgroup analyses, meta-regression)	N/A
	15d	If quantitative synthesis is not appropriate, describe the type of summary planned	15
Meta-bias(es)	16	Specify any planned assessment of meta-bias(es) (such as publication bias across studies, selective reporting within studies)	14-15
Confidence in cumulative evidence	17	Describe how the strength of the body of evidence will be assessed (such as GRADE)	14-15

*** The copyright for PRISMA-P (including checklist) is held by the PRISMA-P Group and is distributed under a Creative Commons Attribution Licence 4.0.**
From: Shamseer L, Moher D, Clarke M, Ghersi D, Liberati A, Petticrew M, Shekelle P, Stewart L, PRISMA-P Group. Preferred reporting items for systematic review and meta-analysis protocols (PRISMA-P) 2015: elaboration and explanation. BMJ. 2015 Jan 2;349(jan02 1):g7647.

For peer review only

Appendix 1: Enhancing transparency in reporting the synthesis of qualitative research: the ENTREQ statement

No.	Item	Guide and description	Reported on Page
1	Aim	State the research question the synthesis addresses.	6-7
2	Synthesis methodology	Identify the synthesis methodology or theoretical framework which underpins the synthesis, and describe the rationale for choice of methodology (e.g. meta-ethnography, thematic synthesis, critical interpretive synthesis, grounded theory synthesis, realist synthesis, meta-aggregation, meta-study, framework synthesis).	7-14
3	Approach to searching	Indicate whether the search was pre-planned (comprehensive search strategies to seek all available studies) or iterative (to seek all available concepts until they theoretical saturation is achieved).	9
4	Inclusion criteria	Specify the inclusion/exclusion criteria (e.g. in terms of population, language, year limits, type of publication, study type).	10-11
5	Data sources	Describe the information sources used (e.g. electronic databases (MEDLINE, EMBASE, CINAHL, psycINFO, Econlit), grey literature databases (digital thesis, policy reports), relevant organisational websites, experts, information specialists, generic web searches (Google Scholar) hand searching, reference lists) and when the searches conducted; provide the rationale for using the data sources.	9-10
6	Electronic Search strategy	Describe the literature search (e.g. provide electronic search strategies with population terms, clinical or health topic terms, experiential or social phenomena related terms, filters for qualitative research, and search limits).	9-10 Appendix 1
7	Study screening methods	Describe the process of study screening and sifting (e.g. title, abstract and full text review, number of independent reviewers who screened studies).	10-11
8	Study characteristics	Present the characteristics of the included studies (e.g. year of publication, country, population, number of participants, data collection, methodology, analysis, research questions).	N/A
9	Study selection results	Identify the number of studies screened and provide reasons for study exclusion (e,g, for comprehensive searching, provide numbers of studies screened and reasons for exclusion indicated in a figure/flowchart; for iterative searching describe reasons for study exclusion and inclusion based on modifications t the research question and/or contribution to theory development).	11-13
10	Rationale for appraisal	Describe the rationale and approach used to appraise the included studies or selected findings (e.g. assessment of conduct (validity and robustness), assessment of reporting (transparency), assessment of content and utility of the findings).	11-12

For peer review only

11	Appraisal items	State the tools, frameworks and criteria used to appraise the studies or selected findings (e.g. Existing tools: CASP, QARI, COREQ, Mays and Pope [25]; reviewer developed tools; describe the domains assessed: research team, study design, data analysis and interpretations, reporting).	11-13
12	Appraisal process	Indicate whether the appraisal was conducted independently by more than one reviewer and if consensus was required.	13
13	Appraisal results	Present results of the quality assessment and indicate which articles, if any, were weighted/excluded based on the assessment and give the rationale.	N/A
14	Data extraction	Indicate which sections of the primary studies were analysed and how were the data extracted from the primary studies? (e.g. all text under the headings "results /conclusions" were extracted electronically and entered into a computer software).	14
15	Software	State the computer software used, if any.	14
16	Number of reviewers	Identify who was involved in coding and analysis.	11-13
17	Coding	Describe the process for coding of data (e.g. line by line coding to search for concepts).	14
18	Study comparison	Describe how were comparisons made within and across studies (e.g. subsequent studies were coded into pre-existing concepts, and new concepts were created when deemed necessary).	14
19	Derivation of themes	Explain whether the process of deriving the themes or constructs was inductive or deductive.	14
20	Quotations	Provide quotations from the primary studies to illustrate themes/constructs, and identify whether the quotations were participant quotations of the author's interpretation.	14
21	Synthesis output	Present rich, compelling and useful results that go beyond a summary of the primary studies (e.g. new interpretation, models of evidence, conceptual models, analytical framework, development of a new theory or construct).	14-15

Extracted from: Tong A, Flemming K, McInnes E, et al. Enhancing transparency in reporting the synthesis of qualitative research: ENTREQ. ***BMC Medical Research Methodology*** **2012;12(1):181. doi: 10.1186/1471-2288-12-181 1**

For peer review only

BMJ Open

What are the experiences of seeking, receiving and providing FGM-related healthcare? Perspectives of health professionals and women / girls who have undergone FGM; Protocol for a systematic review of qualitative evidence

For peer review only

Journal:	*BMJ Open*
Manuscript ID	bmjopen-2017-018170.R2
Article Type:	Protocol
Date Submitted by the Author:	13-Oct-2017
Complete List of Authors:	Evans, Catrin; University of Nottingham School of Health Sciences, School of Health Sciences<br>Tweheyo, Ritah; University of Nottingham School of Health Sciences, School of Health Sciences<br>McGarry, Julie; University of Nottingham,<br>Eldridge, Jeanette; University of Nottingham School of Health Sciences, Research and Learning Services,<br>McCormick, Carol; Queen's Medical Centre<br>Nkoyo, Valentine; Mojatu Foundation<br>Higginbottom, Gina; University of Nottingham School of Health Sciences
**Primary Subject Heading**:	Evidence based practice
Secondary Subject Heading:	Public health, Evidence based practice, Qualitative research, Health services research, Health policy
Keywords:	Female Genital Mutilation/Cutting, qualitative evidence synthesis, immigrant health, health professional perspectives, systematic review, experience of migrant women and girls

SCHOLARONE™
Manuscripts

What are the experiences of seeking, receiving and providing FGM-related healthcare? Perspectives of health professionals and women / girls who have undergone FGM; Protocol for a systematic review of qualitative evidence

Authors:
Dr. Catrin Evans (**corresponding author**)
Associate Professor, and Director of Global Engagement
School of Health Sciences, University of Nottingham, Queens Medical Centre, Nottingham, NG7 2HA. Email: Catrin.Evans@nottingham.ac.uk
orcid.org/0000-0002-5338-2191

Dr. Ritah Tweheyo
Research Fellow, School of Health Sciences, University of Nottingham, Queens Medical Centre, Nottingham, NG7 2HA. Email: Ritah.Tweheyo@nottingham.ac.uk
orcid.org/0000-0002-4609-7253

Dr. Julie McGarry
Associate Professor and Chair of the Domestic Violence and Abuse Integrated Research Group, School of Health Sciences, University of Nottingham, NG7 2HA
orcid.org/0000-0002-7629-2447

Jeanette Eldridge
Senior Research Librarian- Medicine & Health Sciences, Library Research & Learning Services, University of Nottingham, NG7 2RD
orcid.org/0000-0001-7341-4587

Carol McCormick
FGM Specialist Midwife, Nottingham University Hospital Trust

Valentine Nkoyo
Director- Mojatu Foundation, Founder of Nottingham Community FGM Steering Group and FGM Survivors Club

Prof. Gina Marie Awoko Higginbottom MBE,
The Mary Seacole Professor of Ethnicity and Community Health
School of Health Sciences, University of Nottingham Queens Medical Centre, Nottingham, NG7 2HA
orcid.org/0000-0001-8851-0985

Word Count: 3,443 words

For peer review only - http://bmjopen.bmj.com/site/about/guidelines.xhtml

For peer review only

Abstract

Introduction
Female Genital Mutilation (FGM) is an issue of global concern. High levels of migration mean that healthcare systems in higher-income western countries are increasingly being challenged to respond to the care needs of affected communities. Research has identified significant challenges in the provision of, and access to, FGM-related healthcare. There is a lack of confidence and competence among health professionals in providing appropriate care, suggesting an urgent need for evidence-based service development in this area. This study will involve two systematic reviews of qualitative evidence to explore the experiences, needs, barriers and facilitators to seeking and providing FGM-related healthcare in high income (OECD) countries, from the perspectives of: (1) women and girls who have undergone FGM and (2) health professionals.

Review methods
Twelve databases including MEDLINE, EMBASE, PsycINFO, ASSIA, Web of Science, ERIC, CINAHL, and POPLINE will be searched with no limits on publication year. Relevant grey literature will be identified from digital sources and professional networks.

Two reviewers will independently screen, select and critically appraise the studies. Study quality will be assessed using the JBI-QARI appraisal tool. Findings will be extracted into NVivo software. Synthesis will involve inductive thematic analysis, including in-depth reading, line by line coding of the findings, development of descriptive themes and re-coding to higher level analytical themes. Confidence in the review findings will be assessed using the GRADE-CERQual approach. Findings will be integrated into a comprehensive set of recommendations for research, policy and practice.

Dissemination
The syntheses will be reported as per the ENTREQ statement. Two reviews will be published in peer-reviewed journals and an integrated report disseminated at stakeholder engagement events.

Registration
The study is registered with the international prospective register of systematic reviews (PROSPERO): CRD42015030001:2015 and CRD42015030004: 2015.

Funder
The National Institute Health Research (NIHR) Health Service Research and Delivery Programme (NIHR 1115/137/04).

The researchers have no conflicts of interest.

For peer review only

Strengths and Limitations of this Study:

- Will illuminate health professional and organisational factors that influence choices and behaviour on providing FGM-care and following FGM management protocols in a range of healthcare settings (not just maternity settings).
- Will develop an in-depth understanding of women's/girl's care seeking choices, barriers and experiences across the life course (not just maternity settings).
- Will synthesize research in similar high-income contexts so that findings can be directly translated into interventions and service initiatives across OECD countries that share similar challenges in service provision and uptake.
- This is a participatory collaborative project that involves community representatives from identification of the initial questions through to dissemination.
- The systematic reviews will only include qualitative empirical evidence. Hence, a limitation is that opinion pieces, editorials and case studies of personal or professional experiences will be excluded.

Key words:
FGM, Female Genital Mutilation/Cutting, systematic review, qualitative evidence synthesis, migrant women and girls, FGM-related healthcare, health professional perspectives, immigrant health, OECD countries.

For peer review only

Introduction

Female Genital Mutilation (FGM) refers to all procedures that involve the partial or total removal of the external female genitalia or other injury to the female genital organs for non-medical reasons [1]. The practice is illegal in most countries and is an internationally recognised violation of the rights of women and girls [2]. FGM is associated with significant negative physical, psychological and sexual health sequelae [3]. In the immediate and short term, these can include shock, infection, urinary retention or injury to other tissues (e.g. vaginal fistulae) [4 5]. In the longer term, they can include psychological problems, post-traumatic stress disorder, painful intercourse and other sexual problems, relationship problems, chronic pain, chronic infections, infertility and complications in childbirth [1 5]. It is essential therefore that affected women and girls have access to high-quality services that can identify and meet these multiple complex health needs, and that include mental as well as physical healthcare provision [1 6 7].

There are 30 countries where FGM is traditionally practised, with over 200 million women and girls affected worldwide, mainly from Africa and parts of Asia [2]. However, increased migration means that FGM is now regularly seen within health services in higher income ‘receiving’ countries where refugees and migrants from practising countries have settled [2 8]. It has been estimated that over half a million women and girls residing in European Union countries are FGM survivors [8]. Within the UK, it is thought that approximately 137,000 women and girls living in England and Wales have undergone FGM [9], and that, since 2008, women with FGM make up approximately 1.5% (nearly 11,000) of all maternity episodes [9]. Hence, health services and health professionals in receiving countries are increasingly being challenged to develop appropriate services and to care for women/girls who have undergone FGM in a culturally sensitive manner [6 10]. Migrant’s access to healthcare in these countries can be problematic however, due to a range of socio-economic, legal, language and cultural barriers [11 12]. The ways in which these factors intersect to affect appropriate care delivery for women/girls who have undergone FGM – a particularly sensitive and personal issue - is currently unclear.

Existing evidence on women’s and health professionals’ experiences around FGM is primarily orientated to maternity care delivery, with much less attention devoted to care delivery in other settings, or for other problems or at other stages of the life course [1 13]. The available evidence from women however highlights a number of concerns, including not knowing about specialist services, feeling unable to talk about FGM, feeling judged and

For peer review only - http://bmjopen.bmj.com/site/about/guidelines.xhtml

experiencing fear, pain and isolation [14-22]. Likewise, evidence from health professionals indicates that they lack confidence and competence in caring for, and talking about, FGM [10 23-26]. Interestingly, even where training has been given and where clear care pathways and protocols exist, problems may still remain. For example, a recent study of FGM management in a large London maternity unit found that, in spite of the existence of guidelines and training, clinical care for women/girls with FGM was sub-optimal [27]. The maternity unit had access to a FGM specialist service, but 41% of women with FGM were not identified until they arrived in the labour ward. Hence, even though a specialist service existed, it was not being optimally used to benefit women with FGM, and a significant percentage of opportunities were missed to provide women with specialist care. Similar findings were reported from a study in a maternity unit in Switzerland where, in spite of staff training and the existence of clear guidelines, FGM was correctly identified and managed in only 34 (26.4%) of 129 cases reviewed [28]. The reasons for this lack of adherence to protocols are unclear however, and there is a need to explore in more depth how organizational and personal factors may influence health professionals' views and behaviour in this area [10 24 25 29-36].

In the UK, a range of policies and protocols around FGM prevention and care have been developed over the last decade. These set out safeguarding procedures to mitigate risk of FGM and recommend a multi-agency approach to service development [6 37]. In the UK, these include; a declaration to end FGM in 2014, the establishment of a National FGM Prevention Programme, development of Inter-Collegiate Guidelines [38-40], mandatory recording using the SCCI2026 FGM Enhanced Dataset Information Standard, the Serious Crime Act 2015, and mandatory reporting of girls at risk of FGM [41]. Many other OECD countries have similar laws barring FGM, including direct prosecution of suspects continuing the practice [37 42]. All EU countries consider FGM a crime of serious bodily harm, with legal measures to safeguard minors, and professional provisions for disclosure and duty to report [37]. Approaches to prosecution are varied, resulting in calls for efforts to improve and correct the inadequate care of women and girls who have undergone FGM [43], specifically for multidisciplinary approaches [13 44]. It is currently unknown how these legal and regulatory provisions impact upon on the experience of seeking and providing healthcare from patients' and health professionals' perspectives.

Much of the existing body of research around FGM relates to understanding the practice of FGM [45], the prevention of FGM [46 47] and the psycho-social and clinical consequences of

For peer review only - http://bmjopen.bmj.com/site/about/guidelines.xhtml

FGM [3 5 48 49]. Evidence on the effectiveness of FGM specific interventions or models of care is currently lacking [50 51], as concluded by a series of 10 systematic reviews published by WHO in a recent journal supplement [52]. In the absence of high quality evidence on the clinical or cost-effectiveness of different types of service provision, decision making around FGM services must be informed by other forms of evidence [53]. Hence, this project proposes to bring together existing qualitative evidence on lay and professional experiences of, and perspectives on, FGM care, and to relate this to service development in higher income (receiving) countries. In doing so, it will provide a vital resource for evidence-informed FGM-related service development and training. Qualitative research can provide insights into factors influencing service acceptability, appropriateness and meaningfulness, and on micro and macro level organizational and contextual issues that influence service use and delivery [54-56]. Therefore, the proposed qualitative evidence syntheses around FGM will generate rich data and deep understanding with which to inform new service initiatives and the content and structure of staff training resources [57 58]. The above mentioned WHO systematic review series [50] included 3 qualitative reviews, but these were focused on evidence related only to specific interventions, rather than healthcare experiences more generally, and hence identified a very limited number of studies [59-61].

There are a number of systematic reviews related to FGM healthcare that have been published to date, but all have taken a multi-context (or 'lumping') approach to the evidence [13 50 62-64], by including research from high and low-income settings across the world. Many key themes from these reviews therefore are drawn from evidence from very different contexts (see [52]) and are not easily transferable to a high-income setting. These countries generally have strong, well-resourced public health systems and FGM is primarily found within their migrant populations [2]. FGM care and its challenges in these settings, will therefore, be linked to other challenges around providing care for migrant populations (such as lack of familiarity with cultural norms, systems or communication issues [11]). This is a very different situation compared with countries where health systems are weak, where FGM is more prevalent, where health providers may have greater exposure to, and indeed, may even be complicit in, FGM (i.e. medicalisation of FGM [65]). In order to address this shortcoming, the proposed systematic reviews of women's and health professionals' experiences will focus exclusively on evidence from high income OECD contexts.

For peer review only - http://bmjopen.bmj.com/site/about/guidelines.xhtml

Aim

The aim of this review will be two-fold: to understand the experiences, needs, barriers and facilitators around seeking and providing FGM-related care in OECD countries from the perspectives of i) women and girls who have undergone FGM, and ii) health professionals. Hence, this will involve 2 reviews of qualitative evidence:

Review 1: ***To explore the experiences of FGM-related healthcare in OECD countries across the life course for women and girls who have undergone FGM.***

Objectives:

From the perspective of women and girls who have undergone FGM: -

i. to illuminate factors that influence FGM-related healthcare seeking and access to health services across the lifecourse,
ii. to explore how quality of care is perceived and experienced in different healthcare settings and with different groups of healthcare professionals,
iii. to characterise and explain elements of service provision considered important for the provision of acceptable and appropriate healthcare,
iv. to describe factors perceived to influence open discussion and communication around FGM (including prevention) with health professionals.

Review 2: ***To explore the views on, and experiences of, all cadres of health professionals in providing care across the lifecourse in OECD countries for women/girls who have undergone FGM.***

Objectives:

From the perspective of health professionals:

i. to explore how quality of care for women/girls who have undergone FGM is perceived in different healthcare settings and amongst different professional groups,
ii. to characterise and explain elements of service provision considered important for the provision of high quality care to women/girls who have undergone FGM,
iii. to illuminate factors perceived to facilitate or hinder appropriate provision of care for women and girls who have undergone FGM,

For peer review only - http://bmjopen.bmj.com/site/about/guidelines.xhtml

iv. to identify processes and practices perceived to influence open discussion and communication around FGM (including prevention) with women/girls from affected communities.

Methodology and Methods

The review methodology uses a participatory approach in which its aims and objectives were generated together with community organisations working in the field of FGM who continue to be involved as co-investigators and advisors [66]. The project originated from a request by a community organisation run by, and working with, FGM-affected women, to explore women's and health professionals' experiences in the healthcare encounter related to FGM. The review team and advisory group includes members of FGM-affected communities and their perspectives will be integrated into project activities at every stage, for example, in identifying relevant grey literature, contributing to interpretation of key themes, helping to formulate recommendations and helping to access professional and community networks to aid dissemination and community engagement.

The reviews seek to identify insights about lay/health professional experiences of FGM-related healthcare and perceived appropriateness and acceptability of services. These are questions best answered by qualitative research [54 55] – hence the specific focus on qualitative evidence. There are many possible approaches to qualitative evidence synthesis, with most discussions in this area characterizing the different types along a continuum between aggregation and interpretation [67]. Where the purpose of a synthesis is to generate new theoretical insights, a highly interpretive approach such as meta-ethnography may be most suitable, informed by an idealist epistemological stance [68 69]. However, where the purpose is to inform policy or practice a more aggregative or thematic approach informed by a realist epistemology is often advocated [69]. The latter is also suggested in cases where the existing evidence is likely to be descriptive (as in much health services research) rather than highly theoretical or conceptual [57]. An initial scoping of the literature suggests that this is the case for the proposed syntheses. A thematic synthesis approach involves using thematic analysis techniques to identify key concepts/themes within primary research studies [70 71]. Synthesis involves an iterative and inductive process of grouping themes into overarching categories and exploring the similarities, differences and relationships between them [57 58]. Thematic synthesis explicitly aims to move beyond generating a list of descriptive themes (as would be the case in meta-aggregation) in order to identify new, higher order, analytical insights that can contribute to new understandings of a phenomenon [57]. Review

For peer review only - http://bmjopen.bmj.com/site/about/guidelines.xhtml

recommendations however are clearly formulated to inform policy and practice and to identify research gaps. As such, it is considered the most suitable approach for the two proposed systematic reviews [72].

The findings of the two reviews will be reported separately and then, where appropriate, integrated (Fig.1) to enable greater understanding of key issues or concepts when presented from multiple perspectives. The findings will be brought together into a comprehensive set of recommendations for service development, community engagement, health professional education and future research.

Approach to searching

The project will implement a comprehensive search strategy to gather all available and accessible studies, including peer reviewed articles and grey literature. Primary research articles in the form of journal papers, research/evaluation reports, theses and dissertations will be collected. Reference lists from primary papers and key reviews will be hand searched to identify additional papers. There will be no language or date limits.

Data Sources

Searching will be done in at least 12 databases to include Medline (Ovid), Embase, Scopus, PsycINFO, Web of Science, ASSIA, CINAHL, ERIC, MIDIRS using a detailed search strategy (Appendix 1). Additional searches will be carried out in relevant indexes such as POPLINE, grey literature databases including: British library, NICE evidence services, index to theses, Networked Digital Library of Theses and Dissertations (NDLTD), ProQuest and other accessible digital thesis and dissertation repositories.

Search Strategy

The search strategy was developed from an initial scoping search undertaken to establish relevant search terms and potential databases and to develop a robust integrated, but specific and sensitive strategy. It is vital that we have a comprehensive search strategy because of known poor indexing of qualitative studies. Therefore, the scoping search will identify records which will be cross checked for indexing, together with FGM reports and guidelines from WHO to ensure correct terms and FGM-related terms are captured. The Librarian (JE) together with RT will develop and conduct all the searches. The

For peer review only

comprehensive search will be tailored to each of the listed databases for both peer reviewed and grey literature and results reported in a flowchart following PRISMA guidance.

No limits will be placed on language or publication year in the search strategy. We will include articles available from the start of database indexing to present (up to 31st December 2017).The scoping search will run from April to June 2017, and the expanded search will run from July to October 2017. We will set up automatic search alerts and updates until 31st December 2017. Electronic searches are summarised in Fig 2 and an initial detailed search strategy is attached in Appendix 1.

Screening and Eligibility

All returned results from the searches will be entered into the EndNote reference manager programme. Screening will be undertaken independently by 2 reviewers using title and abstract, and studies selected if they meet the a-priori selection criteria (see table 1). In case of disagreement, there will be recourse to a 3rd reviewer when required.

The inclusion criteria are:

- **Population:** Women and girls who have undergone FGM, and their experience of FGM-related healthcare, or healthcare professionals or students involved in the care of women and girls that have undergone FGM.
- **Phenomenon of Interest:** Experiences of seeking, receiving or providing FGM-related healthcare across the life course.
- **Context or Setting:** Studies conducted in OECD/high-income countries as described by the World Bank (List attached in Appendix 1).
- **Study type:** Any type of qualitative study and any type of mixed methods study that reports qualitative findings.
- **Search Limits:** No language or date limits. The abstracts of articles not in English will be translated and assessed. Those that meet the screening criteria will then be professionally translated into English for appraisal and data extraction.
- **Exclusion:** Any papers or research that was not undertaken on women and girls who have undergone FGM, or healthcare professionals, or not in OECD countries. Studies will be excluded if they do not describe FGM-related experiences of healthcare or provision of healthcare.

For peer review only - http://bmjopen.bmj.com/site/about/guidelines.xhtml

For peer review only

Table 1: *Screening Checklist*

Screening Criteria	Yes	No	Unsure
Does the study report findings related to……………… • Experiences of seeking and receiving **healthcare** related to FGM? • Experiences of providing **healthcare** to women/girls who have undergone FGM?			
Is the study conducted in an OECD country?			
Is it a qualitative study? Or Does it report qualitative findings from a mixed methods study?			
Are the participants………………… • Women or girls who have undergone FGM? • Health professionals?			

Quality Appraisal of Studies:

The role of critical appraisal in qualitative evidence synthesis is contested and there is lack of agreement over: (i) the appropriateness of excluding studies, (ii) the potential impact (or not) of excluding eligible papers on review outcomes, and, (iii) the criteria on which quality should be established [68 73-75]. For these reasons, the project will adopt an inclusive approach to critical appraisal, using the appraisal process to enable an in-depth understanding of each paper and to facilitate a critical, questioning approach to the study findings [76]. Studies will not be excluded on the basis of quality, rather, the quality assessment will be used: (i) to judge the relative contribution of each study to the overall synthesis, and, (ii) to assess the methodological rigour of each study as part of a process of assessing confidence in the review findings [77]. The quality of included studies will be assessed independently by 2 reviewers using the Joanna Briggs Institute Qualitative Assessment and Review Instrument (JBI-QARI) [78 79]. This tool has been found to be more coherent and more sensitive to assessment of validity than other commonly used tools [75]. An extract summary of the appraisal items is listed in Table 2.

Data extraction

Data extraction will primarily be undertaken by one reviewer with a 2nd and 3rd reviewer each checking a random sample of the articles for completeness in extraction. Data will be extracted using a modified JBI data extraction form (Table 3), to include details of the phenomenon of interest, population, context, study methodology, methods, and findings of significance to the review objectives [78 80]. If studies lack relevant detail, authors will be contacted to provide further information. The 'results/findings' sections from each study will

For peer review only - http://bmjopen.bmj.com/site/about/guidelines.xhtml

be entered into NVivo® for further analysis and synthesis. There is on-going debate over what constitutes a 'finding' in qualitative research and how to differentiate between findings - i.e. the study authors' analysis/interpretation of the primary data and other inferences or conclusions made by the authors [72 81-83]. In this review, we will follow the approach advocated by Thomas & Harden [57] in which the 'results/findings' section of each paper will be entered into NVivo for detailed thematic analysis (described below). However, if, upon reading a paper, it becomes clear that a finding is reported elsewhere (e.g. in the abstract or discussion section) this excerpt will also be extracted into NVivo.

Where a paper presents findings on both women's *and* health professional's experiences, the relevant sections will be extracted for each review separately where possible.

Synthesis

Analysis will be conducted by 2 reviewers in consultation with the research team. We shall carry out an inductive-thematic analysis for each review [57], conducted in 4 phases: (i) in-depth reading and immersion in the data, (ii) coding of findings that are connected to FGM-related healthcare, (iii) re-coding to higher level themes and (iv) interpretive synthesis. If appropriate, the latter stage may involve the development of models or frameworks to identify and display relationships between, and patterns within, the analytical themes. Where possible, the analytical themes will be formulated as 'directive' findings indicating clear messages and/or suggesting clear lines of action for policy and practice [78].

Assessment of Confidence

The review findings will be assessed using the GRADE-CERQUAL guidelines [77]. The assessment of confidence in the evidence for an individual review finding considers four elements: (i) methodological limitations (the extent to which there are problems in the design or conduct of primary studies that contributed to evidence of a review finding); (ii) relevance (the extent to which the body of evidence from the primary studies supporting a review finding is applicable to the context specified in the review question); (iii) coherence (whether the finding is well grounded in data from the primary studies and can provide a convincing explanation for pattern found in the data); and (iv) adequacy of data (an overall determination of the degree of richness and quantity of data supporting a review finding) [77:2-4]. Based on the assessment, each review finding will be assigned one of four levels of confidence: high, moderate, low and very low.

For peer review only - http://bmjopen.bmj.com/site/about/guidelines.xhtml

***Table 2**: Extracted JBI Quality Appraisal Tool*

Summary JBI Qualitative Assessment and Review Instrument (QARI)													
**Record details / Full reference**	Is there congruity between the stated philosophical perspective and the research methodology?	Is there congruity between the research methodology and the research question or objectives?	Is there congruity between the research methodology and the methods used to collect data?	Is there congruity between the research methodology and the representation and analysis of data?	Is there congruity between the research methodology and the interpretation of results?	Is there a statement locating the researcher culturally or theoretically?	Is the influence of the researcher on the research, and vice-versa, addressed?	Are participants, and their voices, adequately represented?	Is the research ethical according to current criteria or, is there evidence of ethical approval by an appropriate body?	Do the conclusions drawn in the research report flow from the analysis, or interpretation, of the data?	**Score**	Seek further info	Comments (Including reason for exclusion)
	**Q1**	**Q2**	**Q3**	**Q4**	**Q5**	**Q6**	**Q7**	**Q8**	**Q9**	**Q10**	/10		

review only

For peer review only - http://bmjopen.bmj.com/site/about/guidelines.xhtml

Table 3: *Data Extraction tool to include all the results and findings sections of each included study*

Modified JBI data extraction form for FGM Systematic Review											
Study (Name and Authors)	Purpose / Phenomena of interest	Methodology (type, theory / framework)	Methods (type, recruitment and sampling)	Setting	Geographical (Country and Region)	Cultural	Participants (Age, relevant number, sample)	Data analysis	Findings/ Results	Authors Conclusion	Reviewers Comments

iew only

For peer review only - http://bmjopen.bmj.com/site/about/guidelines.xhtml

Presenting and reporting the review

Each review will be reported in accordance with the ‘Enhancing Transparency in Reporting the Synthesis of Qualitative Research’ (ENTREQ) statement [84], appropriate for qualitative evidence synthesis, which consists of 21 items- see Table 4.

Table 4: *Summary of the 21 items from the ENTREQ Statement*

No	Item
1	Aim
2	Synthesis methodology
3	Approach to searching
4	Inclusion criteria
5	Data sources
6	Electronic Search strategy
7	Study screening methods
8	Study characteristics
9	Study selection results
10	Rationale for appraisal
11	Appraisal items
12	Appraisal process
13	Appraisal results
14	Data extraction
15	Software
16	Number of reviewers
17	Coding
18	Study comparison
19	Derivation of themes
20	Quotations
21	Synthesis output

For peer review only

Dissemination

The participatory collaborative approach to this qualitative evidence synthesis project will enhance the multidisciplinary interpretation of findings in order to inform stakeholders and the development of FGM-care initiatives for improved confidence and quality of service provision to women and girls who have undergone FGM.

The 2 qualitative systematic reviews will be published separately in open-access, peer reviewed, international journals and an integrated report compiled and widely disseminated to FGM stakeholders through stakeholder engagement events, round-table discussions with different stakeholder groups, and tailored materials for each of the stakeholder groups

For peer review only - http://bmjopen.bmj.com/site/about/guidelines.xhtml

(academic, health professionals, policy makers/commissioners, FGM communities and community organisations). We shall share the review findings report in the second quarter of 2018, using pre-existing contacts with the WHO Europe FGM group network and through professional networks.

Conflict of interest

The authors declare no competing interests in the development of this manuscript.

Acknowledgements

This systematic review is funded under Project: ***Improving Care for Women and Girls who have undergone Female Genital Mutilation (FGM): Qualitative Evidence Synthesis***. Funded by the National Institute Health Research (NIHR) Health Service Research and Delivery Programme (NIHR 1115/137/04).

Author Contributions

CE conceptualised the project and prepared the project documents as PI in collaboration with the team members GH, JM, CC, and VN.
JE developed the search strategy and will carry out the searches
RT prepared the manuscript with input from team members.
All team members reviewed and approved this manuscript.

References

1. World Health Organisation. WHO guidelines on the management of health complications from Female Genital Mutilation. Geneva: WHO, 2016.
2. United Nations Children's Fund. Female Genital Mutilation/Cutting: A Global Concern. New York: UNICEF, 2016.
3. World Health Organisation. A Systematic Review of the Health Complications of Female Genital Mutilation Including Sequelae in Childbirth. Geneva: World Health Organisation, 2000.
4. Berg RC, Underland V, Odgaard-Jensen J, et al. Effects of female genital cutting on physical health outcomes: A systematic review and meta-analysis. *BMJ Open* 2014;4(11):no pagination.
5. Berg RC, Denison E, A. F. Psychological, social and sexual consequences of female genital mutilation/cutting (FGM/C): a systematic review of quantitative studies. Centre for Reviews and Dissemination. Oslo: Norwegian Knowledge Centre for the Health Services (NOKC), 2010.
6. HM Government. Multi-agency statutory guidance on Female Genital Mutilation. In: Department for Education, Department of Health, Home Office, eds.

For peer review only - http://bmjopen.bmj.com/site/about/guidelines.xhtml

https://www.gov.uk/government/publications/multi-agency-statutory-guidance-on-female-genital-mutilation, 2016.
7. Creighton S, Hodes D. Female Genital Mutilation (FGM): Chapter 3: Annual Report of the Chief Medical Officer: The Health of the 51%: Women. In: Department of Health, ed. London: Department of Health, 2015.
8. Van Baelen L, Ortensi L, Leye E. Estimates of first-generation women and girls with female genital mutilation in the European Union, Norway and Switzerland. *The European Journal of Contraception & Reproductive Health Care* 2016;21(6):474-82.
9. Macfarlane A, Dorkenoo E. Prevalence of female genital mutilation in England and Wales: National and local estimates. London: City University, 2015.
10. Degni F, Suominen S, Essen B, et al. Communication and cultural issues in providing reproductive health care to immigrant women: health care providers' experiences in meeting the needs of [corrected] Somali women living in Finland. *Journal of Immigrant and Minority Health* 2012;14(2):330-43. doi: https://dx.doi.org/10.1007/s10903-011-9465-6
11. Sarría-Santamera A, Hijas-Gómez AI, Carmona R, et al. A systematic review of the use of health services by immigrants and native populations. *Public Health Reviews* 2016;37(1) doi: 10.1186/s40985-016-0042-3
12. Suphanchaimat R, Kantamaturapoj K, Putthasri W, et al. Challenges in the provision of healthcare services for migrants: a systematic review through providers' lens. *BMC Health Services Research* 2015;15:390. doi: 10.1186/s12913-015-1065-z
13. Zurynski Y, Sureshkumar P, Phu A, et al. Female Genital Mutilation and cutting: a systematic literature review of health professionals' knowledge, attitudes and clinical practice. *BMC International Health & Human Rights* 2015;15:32.
14. Berggren V, Bergstrom S, Edberg AK. Being different and vulnerable: Experiences of immigrant African women who have been circumcised and sought maternity care in Sweden. *Journal of Transcultural Nursing* 2006;17(1):50-57.
15. Chalmers B, Hashi KO. 432 Somali women's birth experiences in Canada after earlier female genital mutilation. *Birth* 2000;27(4):227-34.
16. Moxey JM, Jones LL. A qualitative study exploring how Somali women exposed to female genital mutilation experience and perceive antenatal and intrapartum care in England. *BMJ Open* 2016;6 (1) (no pagination)(e009846)
17. Vloeberghs E, Van der kwaak A, Knipscheer J, et al. Coping and chronic psychosocial consequences of female genital mutilation in the Netherlands. *Ethnicity & Health* 2012;17(6):677-95.
18. d'Entremont M, Smythe L, McAra-Couper J. The sounds of silence: A hermeneutic interpretation of childbirth post excision. *Health Care for Women International* 2014;35(3):300-19. doi: http://dx.doi.org/10.1080/07399332.2013.838245
19. Hussein E. Women's experiences, perceptions and attitudes of female genital mutilation: the Bristol PEER study: Foundation for Women's Health Research and Development (FORWARD), 2010.
20. Upvall MJ, Mohammed K, Dodge PD. Perspectives of Somali Bantu refugee women living with circumcision in the United States: a focus group approach. *Int J Nurs Stud* 2009;46(3):360-8. doi: 10.1016/j.ijnurstu.2008.04.009
21. Vangen S, Johansen REB, Sundby J, et al. Qualitative study of perinatal care experiences among Somali women and local health care professionals in Norway. *European Journal of Obstetrics & Gynecology & Reproductive Biology* 2004;112(1):29-35. doi: 10.1016/s0301-2115(03)00313-0
22. Recchia N, McGarry J. "Don't judge me": narratives of living with FGM. *Ethnicity and Inequalities in Health and Social Care* 2017;10(1):4-13.
23. Clayton-Hathway K. A pilot evaluation of health services for communities affected by FGM in Oxfordshire: Final report. Oxford: Oxford Against Cutting, 2016.

For peer review only

For peer review only - http://bmjopen.bmj.com/site/about/guidelines.xhtml

24. Kaplan-Marcusan A, Toran-Monserrat P, Moreno-Navarro J, et al. Perception of primary health professionals about female genital mutilation: from healthcare to intercultural competence. *BMC health services research* 2009;9:11. doi: 10.1186/1472-6963-9-11
25. Dawson AJ, Turkmani S, Varol N, et al. Midwives' experiences of caring for women with female genital mutilation: Insights and ways forward for practice in Australia. *Women and Birth* 2015;28(3):207-14. doi: http://dx.doi.org/10.1016/j.wombi.2015.01.007
26. Johansen E. Care for infibulated women giving birth in Norway. *Medical Anthropology Quarterly* 2006;20(4):516-44.
27. Zenner N, Liao LM, Richens Y, et al. Quality of obstetric and midwifery care for pregnant women who have undergone female genital mutilation. *J Obstet Gynaecol* 2013;33(5):459-62. doi: 10.3109/01443615.2013.767785
28. Abdulcadir J, Dugerdil A, Boulvain M, et al. Missed opportunities for diagnosis of female genital mutilation. *International Journal of Gynaecology and Obstetrics* 2014;125(3):256-60. doi: 10.1016/j.ijgo.2013.11.016
29. Cappon S, L'Ecluse C, Clays E, et al. Female genital mutilation: Knowledge, attitude and practices of Flemish midwives. *Midwifery* 2015;31(3):e29-35 1p. doi: 10.1016/j.midw.2014.11.012
30. Fawcett L. Somali refugee women and their U.S. healthcare providers: Knowledge, perceptions and experiences of childbearing. *Dissertation Abstracts International Section A: Humanities and Social Sciences* 2015;75(8-A(E)):No Pagination Specified.
31. Isman E, Mahmoud Warsame A, Johansson A, et al. Midwives' Experiences in Providing Care and Counselling to Women with Female Genital Mutilation (FGM) Related Problems. *Obstetrics and gynecology international* 2013;2013:785148. doi: 10.1155/2013/785148
32. Kaplan-Marcusan A, Del Rio NF, Moreno-Navarro J, et al. Female genital mutilation: perceptions of healthcare professionals and the perspective of the migrant families. *BMC Public Health* 2010;10:193. doi: 10.1186/1471-2458-10-193
33. León-Larios F, Casado-Mejía R. Knowledge, attitudes and professional experiences of midewives in primary health care in Seville on female genital mutilation. *Matronas Profesion* 2014;15(2):56-61 6p.
34. Tamaddon L, Johnsdotter S, Liljestrand J, et al. Swedish health care providers' experience and knowledge of female genital cutting. *Health Care Women Int* 2006;27(8):709-22. doi: http://dx.doi.org/10.1080/07399330600817741
35. Widmark C, Leval A, Tishelman C, et al. Obstetric care at the intersection of science and culture: Swedish doctors' perspectives on obstetric care of women who have undergone female genital cutting. *Journal of Obstetrics and Gynaecology* 2010;30(6):553-58.
36. Widmark C, Tishelman C, Ahlberg BM. A study of Swedish midwives' encounters with infibulated African women in Sweden. *Midwifery* 2002;18(2):113-25.
37. Leye E, Deblonde J, García-Añón J, et al. An analysis of the implementation of laws with regard to female genital mutilation in Europe. *Crime, Law and Social Change* 2007;47(1):1-31. doi: http://dx.doi.org/10.1007/s10611-007-9055-7
38. Royal College of Midwives, Royal College of Nursing, Royal College of Obstetricians and Gynaecologists, et al. Intercollegiate guidelines tackling FGM in the UK: Intercollegiate recommendations for identifying, recording and reporting. London, 2013.
39. Royal College of Nursing. Female genital mutilation: An RCN resource for nursing and midwifery practice (2nd ed). London: RCN, 2015.
40. Royal College of Obstetricians and Gynaecologists. Green-top guideline no.53: Female genital mutilation and its management. London, 2015.
41. Department of Health. Female Genital Mutilation Risk and Safeguarding; Guidance for professionals. London: Crown copyright, 2016.
42. United States. Agency for International D. Female Genital Mutilation / cutting: United States Government's response: [Washington, D.C.], USAID, 2014 Jul 21., 2014:[3] p.

For peer review only - http://bmjopen.bmj.com/site/about/guidelines.xhtml

43. Rouzi AA, Alturki F. Female Genital Mutilation/cutting: an update. *Clinical and Experimental Obstetrics & Gynecology* 2015;42(3):300-3.
44. Creighton SM, Dear J, de Campos C, et al. Multidisciplinary approach to the management of children with female genital mutilation (FGM) or suspected FGM: service description and case series. *BMJ open* 2016;6(2):e010311.
45. Berg RC, Denison E. A Tradition in Transition: Factors Perpetuating and Hindering the Continuance of Female Genital Mutilation/Cutting (FGM/C) Summarized in a Systematic Review. *Health Care for Women International* 2013;34(10):837-59.
46. Varol N, Turkmani S, Black K, et al. The role of men in abandonment of female genital mutilation: a systematic review. *BMC Public Health* 2015;15(1):1034. doi: 10.1186/s12889-015-2373-2
47. Berg RC, Denison EM. A realist synthesis of controlled studies to determine the effectiveness of interventions to prevent genital cutting of girls. *Paediatrics and international child health* 2013;33(4):322-33. doi: 10.1179/2046905513Y.0000000086
48. Mulango P, Hollins-Martin C, McAndrew S. The psychological impact of Female Genital Mutilation-Cutting (FGM-C) on girls-women's mental health a narrative literature review. *Journal of Reproductive and Infant Psychology* 2014;32(5) doi: 10.1080/02646838.2014.949641
49. Berg RC, Underland V. The obstetric consequences of female genital mutilation/cutting: a systematic review and meta-analysis. *Obstetrics & Gynecology International* 2013;2013:496564. doi: 10.1155/2013/496564
50. Sunday-Adeoye I, Serour G. Management of health outcomes of female genital mutilation: Systematic reviews and evidence syntheses. *International Journal of Gynaecology and Obstetrics* 2017;136 Suppl 1:1-2.
51. Berg RC, Taraldsen S, Said MA, et al. The effectiveness of surgical interventions for women with FGM/C: a Systematic Review. *BJOG: An International Journal of Obstetrics and Gynaecology* 2017 doi: 10.1111/1471-0528.14839
52. Stein K, Hindin JM. Management of health outcomes of female genital mutilation: Systematic reviews and evidence syntheses. *Int J Gynecol Obstet* 2017;136,(Supplement 1,)
53. Abdulcadir J, Rodriguez MI, Say L. Research gaps in the care of women with female genital mutilation: An analysis. *British Journal of Obstetrics and Gynaecology* 2015;122(3):294-303. doi: 10.1111/1471-0528.13217
54. Popay J, Williams G. Qualitative research and evidence based healthcare. *Journal of the Royal Society of Medicine* 1998;35(91):32-37.
55. Pope C, Mays N. Reaching the parts other methods cannot reach: an introduction to qualitative methods in health and health services research. *British Medical Journal* 1995(6996):42-45.
56. Medical Research Council. Developing and evaluating complex interventions: new guidance. London 2008.
57. Thomas J, Harden A. Methods for the thematic synthesis of qualitative research in systematic reviews. *BMC Medical Research Methodology* 2009;8:45.
58. Ring N, Ritchie K, Mandava L, et al. A guide to synthesising qualitative research for researchers undertaking health technology assessment and systematic reviews. Edinburgh: NHS Quality Improvement Scotland and University of Stirling, 2011.
59. Smith H, Stein K. Health information interventions for female genital mutilation. *International Journal of Gynaecology & Obstetrics* 2017;136 Suppl 1:79-82.
60. Smith H, Stein K. Surgical or medical interventions for female genital mutilation. *International Journal of Gynaecology & Obstetrics* 2017;136 Suppl 1:43-46.
61. Smith H, Stein K. Psychological and counselling interventions for female genital mutilation. *International Journal of Gynaecology & Obstetrics* 2017;136 Suppl 1:60-64. doi: https://dx.doi.org/10.1002/ijgo.12051

For peer review only - http://bmjopen.bmj.com/site/about/guidelines.xhtml

62. Hannes K, Harden A. Multi-context versus context-specific qualitative evidence syntheses: combining the best of both. *Research Synthesis Methods* 2011;2(4):271-78. doi: 10.1002/jrsm.55
63. Weir MC, Grimshaw JM, Mayhew A, et al. Decisions about lumping vs. splitting of the scope of systematic reviews of complex interventions are not well justified: a case study in systematic reviews of health care professional reminders. *Journal of Clinical Epidemiology* 2012;65(7):756-63. doi: 10.1016/j.jclinepi.2011.12.012 [published Online First: 2012/04/14]
64. Berg RC, Taraldsen S, Said MA, et al. Reasons for and Experiences With Surgical Interventions for Female Genital Mutilation/Cutting (FGM/C): A Systematic Review. *Journal of Sexual Medicine* 2017;14(8):977-90. doi: 10.1016/j.jsxm.2017.05.016
65. Doucet M-H, Pallitto C, Groleau D. Understanding the motivations of health-care providers in performing female genital mutilation: an integrative review of the literature. *Reproductive Health* 2017;14(1):46.
66. Minkler M, Wallerstein N. Community Based Participatory Research. San Francisco: Jossey Bass 2003.
67. Barnett-Page E, Thomas J. Methods for the synthesis of qualitative research: a critical review. *BMC Mecial Research Methodology* 2009;9:59.
68. Toye F, Seers K, Allcock N, et al. 'Trying to pin down jelly'-exploring intuitive processes in quality assessment for meta-ethnography. *BMC Medical Research Methodology* 2013;13(1):46.
69. Paterson B. "It looks great but how do I know it fits?": An introduction to meta-synthesis research. In: Hannes H, Lockwood C, eds. Synthesizing Qualitative Research: Choosing the Right Approach. Oxford: Wiley Blackwell BMJ Books 2012:1-21.
70. Boyatzis R. Transforming qualitative data: thematic analysis and code development. London: Sage 1998.
71. Braun V, Clarke V. Using thematic analysis in psychology. *Qualitative Research in Psychology* 2006;3(2):77-101.
72. Noyes J, Booth A, Hannes K, et al. Supplementary guidance for inclusion of qualitative research in Cochrane systematic reviews of interventions, Version 1: Cochrane Collaboration Qualitative Methods Group 2011.
73. Carroll C, Booth A, Lloyd-Jones M. Should We Exclude Inadequately Reported Studies From Qualitative Systematic Reviews? An Evaluation of Sensitivity Analyses in Two Case Study Reviews. *Qualitative Health Research* 2012;22(10):1425-34.
74. Cohen D, Crabtree B. Evaluative criteria for qualitative research in health care: controversies and recommendations. *Ann Fam Med* 2008;6:4.
75. Hannes K, Lockwood C, Pearson A. A comparative analysis of three online appraisal instruments' ability to assess validity in qualitative research. *Qualitative Health Research* 2010;20(12):1736-43. doi: 10.1177/1049732310378656 [published Online First: 2010/07/31]
76. Hannes K, Pearson A. Obstacles to the Implementation of Evidence Based Practice in Belgium: a Worked Example of Meta-Aggregation. In: Hannes K, Lockwood C, eds. Synthesizing Qualitative Research: Choosing the Right Approach: BMJ Books, Wiley Blackwell 2012:21-41.
77. Lewin S, Glenton C, Munthe-Kaas H, et al. Using qualitative evidence in decision making for health and social interventions: An approach to assess confidence in findings from qualitative evidence syntheses (GRADE-CERQUAL). *Plos Medicine* 2015;12(10):e1001895.
78. Pearson A, Robertson-Malt S, Rittenmeyer L. Synthesizing qualitative evidence. Philadelphia: Lippincott-Joanna Briggs Institute 2011.
79. The Joanna Briggs Institute. Critical Appraisal Checklist for Qualitative Research 2016 [Available from: http://joannabriggs.org/research/critical-appraisal-tools.html.
80. The Joanna Briggs Institute. JBI QARI Data Extraction Form for Interpretive and Critical Research 2016 [Available from: http://joannabriggs.org/assets/docs/jbc/operations/dataExtractionForms/JBC_Form_DataE_IntCrit.pdf.

For peer review only - http://bmjopen.bmj.com/site/about/guidelines.xhtml

81. Sandelowski M, Barroso J. Finding the findings in qualitative studies. *Journal of Nursing Scholarship* 2002;34:213-19.
82. Noyes J, Hannes K, Booth A, et al. Qualitative and Implementation Evidence and Cochrane Reviews, version 1.02a. In: Higgins JA, Green S, eds. Cochrane Handbook for Systematic Reviews of Interventions Version 530 (updated October 2015): The Cochrane Collaboration, 2015.
83. Jordan R, Dainty K, Noyes J, et al. Factors that impact on the use of mechanical ventilation weaning protocols in critically ill adults and children: a qualitative evidence-synthesis. *Cochrane Database of Systematic Reviews* 2016(10)
84. Tong A, Flemming K, McInnes E, et al. Enhancing transparency in reporting the synthesis of qualitative research: ENTREQ. *BMC Medical Research Methodology* 2012;12(1):181. doi: 10.1186/1471-2288-12-181

For peer review only

For peer review only - http://bmjopen.bmj.com/site/about/guidelines.xhtml

Figure legends

Figure 1: Systematic reviews to improve FGM-related care

Figure 2: Search Summary flowchart

Tables:

Table 1: *Screening Checklist*

Table 2: *Extracted JBI Quality Appraisal Tool*

Table 3: *Data Extraction tool to include all the results and findings sections of each included study*

Table 4: *Summary of the 21 items from the ENTREQ Statement*

For peer review only

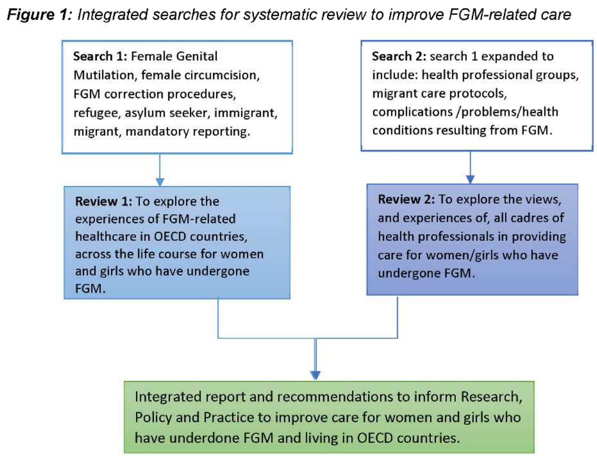

Figure 1: Systematic reviews to improve FGM-related care

209x297mm (300 x 300 DPI)

Figure 2: Search summary flowchart

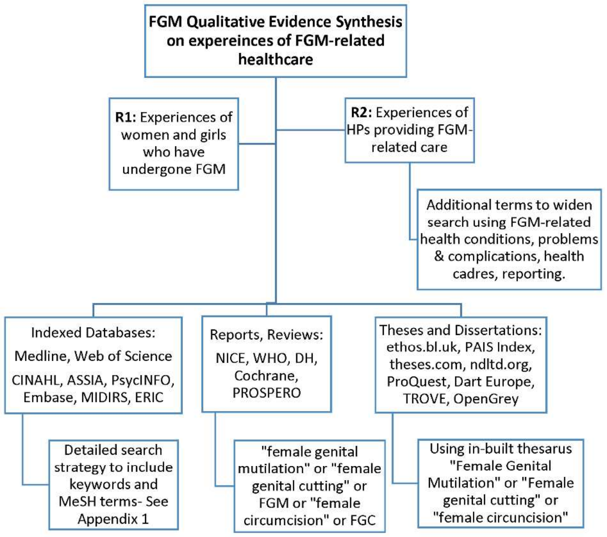

Figure 2: Search summary flowchart

209x297mm (300 x 300 DPI)

Appendix 1: Detailed Search Strategy

The search strategy was developed in order to meet the requirements for both systematic reviews:

- **R1-**To explore the experiences of FGM-related healthcare across the life course for women and girls who have undergone FGM.
- **R2-**To explore the views on, and experiences of, all cadres of health professionals in providing care for women/girls who have undergone FGM.

Search strategy in Ovid Medline

#	Search terms	Notes
1.	(exp Circumcision, Female/ or (exp Genitalia, Female/ and exp Medicine, Traditional/))	
2.	("female genital mutilation" or "female circumcision" or "female genital cutting" or "traditional female genital surgery" or infibulat* or reinfibulat* or defibulat* or clitoridect* or ((((ritual* or traditional* or ceremon* or sociali#ation*) adj3 (practic* or cut or cutting or surg*)) or circumcis* or excis*) adj3 (female* or wom#n or girl* or child* or adolescen*))).mp.	Different FGM-related terms
3.	((FGM or FGC or TFGS or FGMC or "FGM/C" or thara or tahar or khitan or khifad or khafad or megrez or absum or mekhnishab or kutairi or ibi or ugwu or sunna or bondo or sonde or gudiniin or halalays or qodiin or tahoor or bagne or gadja or ganza or "fanadu di mindjer" or niaka or kuyango or "musolula karoola").mp. or (egypt* or sudan* or ethiopi* or somali* or djibouti* or eritrea* or burkin* or guinea* or sierra leone or leonean* or mauritan* or mali or malian*).ti,ab.) adj3 (female* or wom#n or girl* or child* or adolescen*)	Expanded FGM terms (to include country specific name for the practice.
4.	1 or 2 or 3	
5.	"Emigrants and Immigrants"/ or Refugees/ or "Transients and Migrants"/ or "Emigration and Immigration"/ or exp Vulnerable Populations/ or exp Ethnic Groups/ or 31. exp african continental ancestry group/ or exp asian continental ancestry group/	Immigrant populations
6.	Limit 5 to female	
7.	(immigrant* or immigrat* or migrant* or migrat* or emigrant* or emigrat* or emigre* or expat* or ex-pat* or transients or newcomer* or new-comer* or alien* or incomer* or in-comer* or refugee* or (asylum adj seek*) or asylee* or (refused adj3 (asylum* or refugee*)) or (displaced adj person*) or exile* or “new arrival*” or (country adj2 (birth or origin)) or transnational* or foreigner* or (foreign adj (born or citizen* or national* or origin*)) or (non adj (citizen* or native*)) or ((adoptive or naturali#ed) adj (citizen* or resident*)) or overstay* or trafficked or "spousal migrant*" or ethnic* or ethno* or race or racial*).mp. or ((vulnerab* or disadvantag* or minorit*) adj3 (individ* or person* or people* or population* or communit* or group*)).ti,ab.	Expanded immigrant terms commonly used in OECD countries
8.	Limit 7 to female	
9.	("Black and Minority Ethnic" or "Black & Minority ethnic" or BME or BAME or black african*).mp. or (africa* or "middle east*" or asia* or iran* or iraq* or israel* or oman* or "united arab emirat*" or UAE or "saudi arabia*" or palestin* or india* or indonesia* or malaysia* or pakistan* or egypt* or yemen* or sudan* or ethiopi* or somali* or djibouti* or eritrea* or kenya* or uganda* or	Expanded immigrant terms to include countries and populations

	tanzania* or camero?n* or chad* or niger* or benin* or togo* or ghana* or burkin* or ivory coast* or cote d'ivoire or liberia* or guinea* or sierra leone or leonean* or gambia* or senegal* or mauritan* or mali or malian* or "sara subgroup" or ngama).ti,ab.	where FGM is a common practice
10.	Limit 9 to female	
11.	6 or 8 or 10	
12.	exp Community Health Workers/ or exp Volunteers/ or exp Occupational Groups/ or exp Health Personnel/ or exp Midwifery/ or exp General Practitioners/ or exp Physicians/ or exp Nurses/ or exp Nurses, Community Health/ or exp Nurses' Aides/ or exp Nursing Staff, Hospital/ or exp Nursing Staff/	Health professional cadres
13.	(pregnan* or birth* or childbirth* or matern* or gyn#e* or obstetric* or menstru* or labo#r* or vulv* or vagina* or uter*).ti,ab.	Commonly reported in regard to FGM
14.	exp Pregnancy/ or exp Pregnancy Complications/ or exp Pregnancy High Risk/ or exp Obstetric Labor Complications/ or Female Urogenital Diseases/ or exp Menstruation Disturbances/ or exp Genital Diseases, Female/ or exp Vaginal Diseases/ or exp Vulvar Diseases/ or exp Vaginismus/ or exp Vulvodynia/ or exp Dyspareunia/ or exp Vaginitis/ or exp Vulvovaginitis/ or exp Vaginal Fistula/ or exp Rectovaginal Fistula/ or Genital Diseases, Female/ or exp Infertility, Female/	FGM-related conditions, problems, complications
15.	((pelvic* or back* or urolog* or urogenit* or urinat* or genit* or abdomin*) adj3 (poor* or adverse or complicat* or difficult* or disorder* or dysfunction* or disease* or pain* or risk* or danger* or problem* or issue* or concern* or infect* or inflamm*) adj3 (female* or wom#n or girl* or child* or adolescen*)).mp.	
16.	exp Pelvic Inflammatory Disease/ or exp Urologic Diseases/ or exp Pelvic Pain/ or exp Sexual Dysfunctions, Psychological/ or exp Pelvic Infection/ or exp Kidney Diseases/ or exp Urinary Fistula/ or exp Rectal Fistula/ or exp Urinary Bladder Fistula/	
17.	Limit 16 to female	
18.	((anxiet* or anxious* or depress* or self-harm* or self-injur* or suicid*) adj3 (female* or wom#n or girl* or child* or adolescen*)).mp.	Expanded to include mental health conditions
19.	exp Mental Disorders/ or exp Depressive Disorder, Major/ or exp Suicide/ or exp Depression/ or exp Suicidal Ideation/ or exp Depressive Disorder/ or exp Bipolar Disorder/ or exp Suicide, Attempted/ or exp Self-Injurious Behavior/ or exp Substance-Related Disorders/ or exp Social Isolation/	
20.	Limit 19 to female	
21.	((((sex* or viral* or virus* or bacteria*) adj3 diseas*) or HIV or STD* or AIDS) adj3 (female* or wom#n or girl* or child* or adolescen*)).mp.	
22.	exp Sexually Transmitted Diseases, Viral/ or exp Sexually Transmitted Diseases/	
23.	Limit 22 to female	
24	13 or 14 or 15 or 17 or 18 or 20 or 21 or 23	
25.	(AUSTRALIA* or AUSTRIA* or BELGIUM* or belgian* or CANAD* or CHILE* or CZECH* or DENMARK* or danish* or ESTONIA* or FINLAND or finnish or FRANCE or French or GERMAN* or GREECE or greek* or HUNGAR* or ICELAND* or IRELAND or irish or ISRAEL* or ITALY or italian* or JAPAN* or KOREA* or LATVIA* or LUXEMB* or MEXIC* or NETHERLAND* or holland* or dutch or "low countries" or europe* or "NEW ZEALAND*" or NORWAY or	OECD countries

For peer review only

	norwegian* or POLAND or polish or PORTUGAL or portuguese or SLOVAK* or SLOVENIA* or SPAIN or spanish or SWED* or SWITZERLAND or swiss or TURK* or "UNITED KINGDOM" or "UK" or britain or british or england or english or scot* or wales or welsh or UNITED STATES or "USA" or america*).mp.	
26.	randomized controlled trial.pt.	Study types not relevant for qualitative evidence
27.	controlled clinical trial.pt.	
28.	randomized.ab.	
29.	placebo.ab.	
30.	clinical trials as topic.sh.	
31.	randomly.ab.	
32.	trial.ti.	
33.	(animals not (humans and animals)).sh.	
34.	26 or 27 or 28 or 29 or 30 or 31 or 32	
35.	34 not 33	
36.	11 and 12 and 24	
37.	4 and 12	
38.	4 or 36 or 37	
39.	38 and 25	
40.	39 not 35	
41.	40 not 33	

For peer review only

PRISMA-P (Preferred Reporting Items for Systematic review and Meta-Analysis Protocols) 2015 checklist: recommended items to address in a systematic review protocol*

Section and topic	Item No	Checklist item	Reported on Page
**ADMINISTRATIVE INFORMATION**			**1**
Title:			1
Identification	1a	Identify the report as a protocol of a systematic review	1
Update	1b	If the protocol is for an update of a previous systematic review, identify as such	
Registration	2	If registered, provide the name of the registry (such as PROSPERO) and registration number	1
Authors:			1
Contact	3a	Provide name, institutional affiliation, e-mail address of all protocol authors; provide physical mailing address of corresponding author	1
Contributions	3b	Describe contributions of protocol authors and identify the guarantor of the review	16
Amendments	4	If the protocol represents an amendment of a previously completed or published protocol, identify as such and list changes; otherwise, state plan for documenting important protocol amendments	
Support:			
Sources	5a	Indicate sources of financial or other support for the review	2
Sponsor	5b	Provide name for the review funder and/or sponsor	16
Role of sponsor or funder	5c	Describe roles of funder(s), sponsor(s), and/or institution(s), if any, in developing the protocol	16
**INTRODUCTION**			**4-7**
Rationale	6	Describe the rationale for the review in the context of what is already known	4-5
Objectives	7	Provide an explicit statement of the question(s) the review will address with reference to participants, interventions, comparators, and outcomes (PICO)	6-7
**METHODS**			**7-14**
Eligibility criteria	8	Specify the study characteristics (such as PICO, study design, setting, time frame) and report characteristics (such as years considered, language, publication status) to be used as criteria for eligibility for the review	10-11
Information sources	9	Describe all intended information sources (such as electronic databases, contact with study authors, trial registers or other grey literature sources) with planned dates of coverage	9
Search strategy	10	Present draft of search strategy to be used for at least one electronic database, including planned limits, such that it could be repeated	9-10
Study records:			7-14
Data management	11a	Describe the mechanism(s) that will be used to manage records and data throughout the review	7-8
Selection process	11b	State the process that will be used for selecting studies (such as two independent reviewers) through each phase of the review (that is, screening, eligibility and inclusion in meta-analysis)	11-14
Data collection process	11c	Describe planned method of extracting data from reports (such as piloting forms, done independently, in duplicate), any processes for obtaining and confirming data from investigators	13-14
Data items	12	List and define all variables for which data will be sought (such as PICO items, funding sources), any pre-planned data assumptions and	10-11

For peer review only

		simplifications	
Outcomes and prioritization	13	List and define all outcomes for which data will be sought, including prioritization of main and additional outcomes, with rationale	14
Risk of bias in individual studies	14	Describe anticipated methods for assessing risk of bias of individual studies, including whether this will be done at the outcome or study level, or both; state how this information will be used in data synthesis	14-15
Data synthesis	15a	Describe criteria under which study data will be quantitatively synthesised	14-15
	15b	If data are appropriate for quantitative synthesis, describe planned summary measures, methods of handling data and methods of combining data from studies, including any planned exploration of consistency (such as I^2, Kendall's τ)	N/A
	15c	Describe any proposed additional analyses (such as sensitivity or subgroup analyses, meta-regression)	N/A
	15d	If quantitative synthesis is not appropriate, describe the type of summary planned	15
Meta-bias(es)	16	Specify any planned assessment of meta-bias(es) (such as publication bias across studies, selective reporting within studies)	14-15
Confidence in cumulative evidence	17	Describe how the strength of the body of evidence will be assessed (such as GRADE)	14-15

*** The copyright for PRISMA-P (including checklist) is held by the PRISMA-P Group and is distributed under a Creative Commons Attribution Licence 4.0.**

From: Shamseer L, Moher D, Clarke M, Ghersi D, Liberati A, Petticrew M, Shekelle P, Stewart L, PRISMA-P Group. Preferred reporting items for systematic review and meta-analysis protocols (PRISMA-P) 2015: elaboration and explanation. BMJ. 2015 Jan 2;349(jan02 1):g7647.

For peer review only

Appendix 1: Enhancing transparency in reporting the synthesis of qualitative research: the ENTREQ statement

No.	Item	Guide and description	Reported on Page
1	Aim	State the research question the synthesis addresses.	6-7
2	Synthesis methodology	Identify the synthesis methodology or theoretical framework which underpins the synthesis, and describe the rationale for choice of methodology (e.g. meta-ethnography, thematic synthesis, critical interpretive synthesis, grounded theory synthesis, realist synthesis, meta-aggregation, meta-study, framework synthesis).	7-14
3	Approach to searching	Indicate whether the search was pre-planned (comprehensive search strategies to seek all available studies) or iterative (to seek all available concepts until they theoretical saturation is achieved).	9
4	Inclusion criteria	Specify the inclusion/exclusion criteria (e.g. in terms of population, language, year limits, type of publication, study type).	10-11
5	Data sources	Describe the information sources used (e.g. electronic databases (MEDLINE, EMBASE, CINAHL, psycINFO, Econlit), grey literature databases (digital thesis, policy reports), relevant organisational websites, experts, information specialists, generic web searches (Google Scholar) hand searching, reference lists) and when the searches conducted; provide the rationale for using the data sources.	9-10
6	Electronic Search strategy	Describe the literature search (e.g. provide electronic search strategies with population terms, clinical or health topic terms, experiential or social phenomena related terms, filters for qualitative research, and search limits).	9-10 Appendix 1
7	Study screening methods	Describe the process of study screening and sifting (e.g. title, abstract and full text review, number of independent reviewers who screened studies).	10-11
8	Study characteristics	Present the characteristics of the included studies (e.g. year of publication, country, population, number of participants, data collection, methodology, analysis, research questions).	N/A
9	Study selection results	Identify the number of studies screened and provide reasons for study exclusion (e,g, for comprehensive searching, provide numbers of studies screened and reasons for exclusion indicated in a figure/flowchart; for iterative searching describe reasons for study exclusion and inclusion based on modifications t the research question and/or contribution to theory development).	11-13
10	Rationale for appraisal	Describe the rationale and approach used to appraise the included studies or selected findings (e.g. assessment of conduct (validity and robustness), assessment of reporting (transparency), assessment of content and utility of the findings).	11-12

For peer review only

11	Appraisal items	State the tools, frameworks and criteria used to appraise the studies or selected findings (e.g. Existing tools: CASP, QARI, COREQ, Mays and Pope [25]; reviewer developed tools; describe the domains assessed: research team, study design, data analysis and interpretations, reporting).	11-13
12	Appraisal process	Indicate whether the appraisal was conducted independently by more than one reviewer and if consensus was required.	13
13	Appraisal results	Present results of the quality assessment and indicate which articles, if any, were weighted/excluded based on the assessment and give the rationale.	N/A
14	Data extraction	Indicate which sections of the primary studies were analysed and how were the data extracted from the primary studies? (e.g. all text under the headings "results /conclusions" were extracted electronically and entered into a computer software).	14
15	Software	State the computer software used, if any.	14
16	Number of reviewers	Identify who was involved in coding and analysis.	11-13
17	Coding	Describe the process for coding of data (e.g. line by line coding to search for concepts).	14
18	Study comparison	Describe how were comparisons made within and across studies (e.g. subsequent studies were coded into pre-existing concepts, and new concepts were created when deemed necessary).	14
19	Derivation of themes	Explain whether the process of deriving the themes or constructs was inductive or deductive.	14
20	Quotations	Provide quotations from the primary studies to illustrate themes/constructs, and identify whether the quotations were participant quotations of the author's interpretation.	14
21	Synthesis output	Present rich, compelling and useful results that go beyond a summary of the primary studies (e.g. new interpretation, models of evidence, conceptual models, analytical framework, development of a new theory or construct).	14-15

Extracted from: Tong A, Flemming K, McInnes E, et al. Enhancing transparency in reporting the synthesis of qualitative research: ENTREQ. ***BMC Medical Research Methodology*** **2012;12(1):181. doi: 10.1186/1471-2288-12-181 1**

For peer review only
